# Organoids in Cancer Research and Regenerative Medicine: Current Status, Challenges, and Future Prospects

**DOI:** 10.1002/mco2.70575

**Published:** 2026-01-05

**Authors:** Ruiyang Li, Yuezhou Wu, Zhu'anzhen Zheng, Fengjin Zhou, Ke Xu, Jiacan Su

**Affiliations:** ^1^ Department of Orthopedics Xinhua Hospital Affiliated to Shanghai Jiao Tong University School of Medicine Shanghai China; ^2^ MedEng‐X Institutes Shanghai University Shanghai China; ^3^ Trauma Orthopedics Center Xinhua Hospital Affiliated to Shanghai Jiao Tong University School of Medicine Shanghai China; ^4^ Institute of Musculoskeletal Injury and Translational Medicine of Organoids Xinhua Hospital Affiliated to Shanghai Jiao Tong University School of Medicine Shanghai China; ^5^ Institute of Translational Medicine Shanghai University Shanghai China; ^6^ National Center for Translational Medicine SHU Branch Shanghai University Shanghai China; ^7^ Department of Orthopedics Honghui Hospital Xi'an Jiao Tong University Xi'an China

**Keywords:** cancer, disease modeling, drug screening, organoids, regenerative medicine

## Abstract

Cancer and tissue regeneration pose great challenges to global health, as cancer treatment is impeded by tumor heterogeneity and therapy resistance, while regenerative medicine is constrained by donor shortages and difficulties in replicating native tissue structures. Organoids, as advanced three‐dimensional multicellular structures derived from stem cells, have emerged as transformative tools in biomedical research. They recapitulate key aspects of native human tissue composition and functions, offering enhanced physiological relevance over traditional models. Therefore, this review aims to highlight the latest advancements in organoid technology within the fields of cancer research and regenerative medicine. We begin by discussing the fundamental aspects of organoid generation, characterization, and application. Furthermore, recent progress in both cancer‐oriented and regeneration‐focused organoids is summarized, with an emphasis on their applications in disease modeling, drug screening, mechanistic analysis, and precision medicine. Based on an extensive review of the literature, the current challenges and future directions in the development and application of organoid models are discussed. As organoid technology continues to evolve, it is anticipated that more high‐quality studies will further advance medical science and foster innovation in personalized therapeutics.

## Graphical abstract

The management of cancer and tissue regeneration remains a substantial challenge to global health. Organoids, which are stem cell‐derived three‐dimensional multicellular structures, are revolutionizing biomedical research. Therefore, this review highlights their applications in cancer research and regenerative medicine, while discussing current challenges and future prospects based on comprehensive literature analysis.

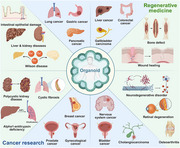

## 1 Introduction

Cancer and tissue regeneration represent two of the most formidable challenges to global human health [1, 2]. Cancer remains a leading cause of mortality worldwide, with tumor heterogeneity and the emergence of therapy resistance posing significant obstacles to effective and personalized treatment [3, 4, 5]. In contrast, the advancement of research in regenerative medicine is often limited by the shortage of donor organs and the complexities of recapitulating native tissue architecture [6]. Traditional disease models, such as two‐dimensional (2D) cell cultures and animal models, have limitations including poor physiological relevance, species differences, and lack of cell–cell interactions [7, 8]. These disadvantages have created a translational gap between preclinical research and clinical outcomes. Consequently, there is an urgent and pressing need for innovative, human‐relevant model systems that can bridge this gap, accelerate drug discovery, and pave the way for personalized therapeutic strategies.

Organoids, as miniaturized, self‐organized three‐dimensional (3D) tissue cultures derived from stem cells, can simulate the structure, function, cellular heterogeneity, and microenvironment of in vivo organs and tissues [9, 10, 11]. The core strengths of organoid technology lie in its retention of patient‐specific characteristics, ability to model tissue complexity, and high‐throughput capacity [12, 13, 14]. Organoids are now widely applied across diverse disease areas, including cancers, neural disorders, infectious diseases, degenerative diseases, and metabolic diseases [15]. They also serve as valuable tools for drug screening, provide models for studying gene‐editing therapies against genetic disorders, and hold promise as potential transplantable tissues to restore organ function [16]. Therefore, organoid‐based disease research is a systematic process encompassing disease modeling, characterization, and mechanistic analysis [17, 18]. Organoids are expected to become powerful tools in translational medicine and precision healthcare, offering a physiologically relevant platform for in‐depth disease research.

**FIGURE 1:**
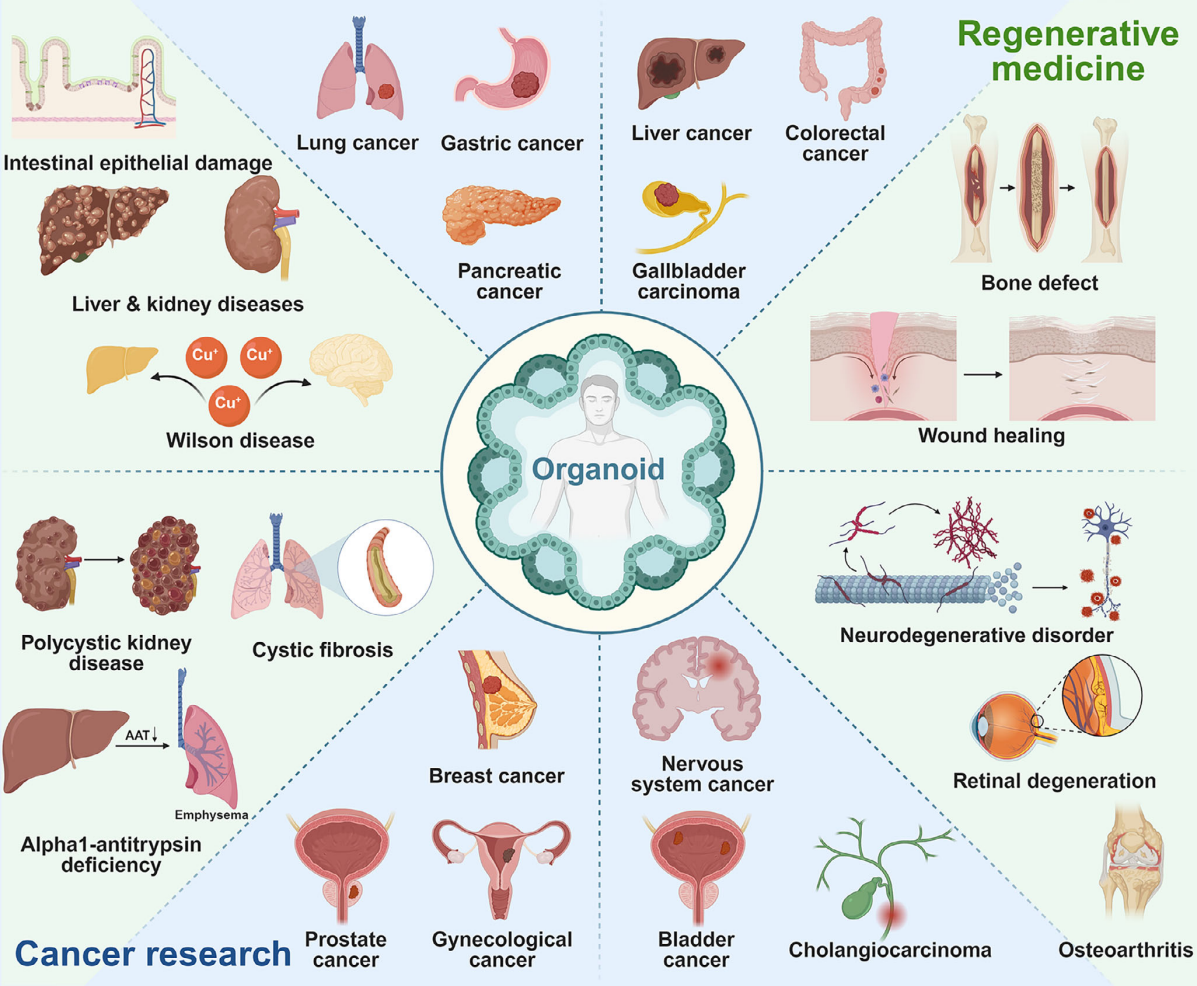
Schematic diagram introducing diseases involved in organoid study in the fields of cancer research and regenerative medicine. Created using BioRender.com.

Therefore, this review will delve into the current applications, key challenges, and future prospects of organoid technology in cancer research and regenerative medicine (Figure 1). We focus primarily on the fundamentals of organoid technology, which include the generation, characterization, validation, and application of organoids. Then, we discuss the current research status of various cancer organoids and regenerative organoids with an emphasis on their applications in disease modeling, drug screening, and mechanistic analysis. Based on a comprehensive insight into organoid research, we assess current progress and discuss key challenges that need to be addressed to create more fully functional organoid disease models. We hope this review will outline future directions to enhance the clinical translation and application of organoid technology in oncology and regenerative medicine.

## 2 Overview of Organoid Technology

**FIGURE 2:**
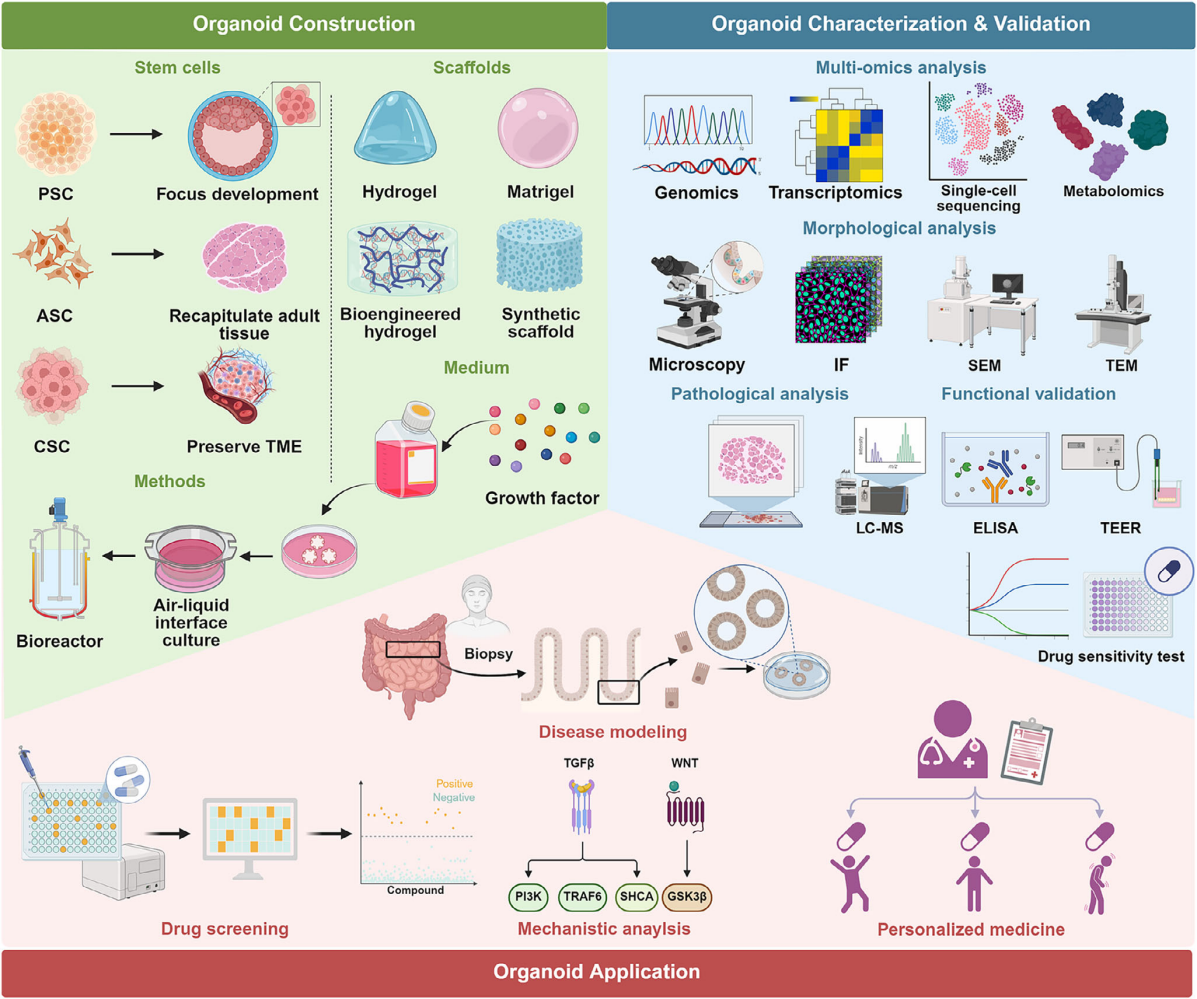
An overview of organoid technology. By mimicking organ structure and function through stem cell‐derived biomimetic culture, organoids have become indispensable tools for disease modeling, drug screening, mechanistic analysis, and precision medicine. Created using BioRender.com.

Organoid technology is advancing so rapidly that the applications of organoids have expanded to various medical fields [19, 20, 21]. Understanding the principles and fundamental techniques of organoid culture is essential for effectively utilizing organoids in research and paves the way for further discussion of their applications and challenges. Here, we introduce the construction, characterization, validation, and application of organoids (Figure 2).

### 2.1 Organoid Construction

Organoids are primarily derived from three cell sources: pluripotent stem cells (PSCs), adult stem cells (ASCs), and cancer stem cells (CSCs) [22, 23, 24, 25]. These cell sources fundamentally determine organoid characteristics and application potential. PSCs include embryonic stem cells and induced PSCs (iPSCs) that can differentiate into cells of any germ layer [26, 27]. Organoids derived from PSCs can model early developmental processes but may lack mature tissue features [28]. Since ASCs are isolated directly from tissues, ASC‐derived organoids can more closely recapitulate adult tissue physiology and pathology. They retain patient‐specific mutations and heterogeneity, which make them ideal for personalized medicine [29]. CSCs are cells enriched from cancer specimens. CSCs‐derived cancer organoids highly preserve the original tumor microenvironment (TME) and drug resistance profiles, serving as powerful tools for cancer research. While 2D cell cultures lack physiological complexity, scaffolds provide a 3D culture space for organoids and concurrently mimic the microenvironment surrounding stem cells [30]. Natural scaffolds, such as basement membrane extracts Matrigel, contain laminin and collagen and can supply bio‐adhesive signals [31]. Matrigel‐embedded 3D organoids from conditionally reprogrammed cells preserve tumor molecular profiles [32]. In pancreatic cancer, these 3D models demonstrated superior clinical correlation over 2D systems for combination regimens such as gemcitabine plus nab‐paclitaxel, evidenced by significantly elevated inhibitory concentration (IC_50_) values indicative of functional drug barriers. This established 3D organoids as translationally faithful preclinical platforms. However, the batch‐to‐batch variability in Matrigel's mechanical and biochemical properties compromises experimental reproducibility and introduces uncertainty [33]. Furthermore, its resistance to physical and biochemical modifications hinders precise control over the microenvironment, limiting the ability to guide specific cellular responses [34]. Hydrogels represent another type of scaffold frequently employed in organoid culture systems [35]. These materials possess mechanical and physical properties that provide a supportive environment for cellular processes. Synthetic biomaterials such as polyethylene glycol (PEG) and gelatin methacryloyl (GelMA) can be cross‐linked to form hydrogels, which offer tunable mechanical characteristics and controllable degradation rates. Moreover, hydrogels fabricated from decellularized extracellular matrix (ECM) exhibit remarkable biomimetic properties, closely emulating the multifaceted functions of native ECM [36]. By preserving tissue‐specific biochemical composition and mechanical cues, decellularized ECM‐based hydrogels create a physiologically relevant microenvironment that is essential for organoid development. Besides the selection of stem cells and scaffolding materials, the establishment of appropriate culture conditions is equally pivotal for successful organoid construction. This involves supplementing the culture medium with specific growth factors to direct differentiation and maturation, followed by incubation under controlled environments such as air–liquid interface systems or advanced bioreactors to support nutrient exchange and 3D tissue development.

### 2.2 Organoid Characterization and Validation

The characterization of organoids aims to demonstrate their high degree of similarity to the source organ or specific tissue in terms of structure, cell types, and molecular levels, which lays the foundation for subsequent functional validation. Comprehensive characterization necessitates synergistic morphological and molecular phenotyping to ensure the full functioning of organoids [37]. Morphological analysis primarily utilizes organoid imaging and histopathological techniques. Brightfield and fluorescence microscopy evaluate overall organoid architecture and identify structural features such as acini or crypts. Confocal microscopy resolves spatial cellular distribution within multilayered organoids. To reveal ultrastructural details like microvilli and tight junctions, scanning or transmission electron microscopy is required. Histological assessment via hematoxylin and eosin staining of paraffin‐embedded sections evaluates architectural similarity to native tissues. Concurrently, immunohistochemistry staining detects lineage‐specific markers within organoids to validate cellular identity. The application of multiomics analysis provides a powerful framework for the in‐depth assessment of organoids [38]. This methodology integrates datasets from the genomic, transcriptomic, and proteomic levels to elucidate intricate molecular networks and construct a dynamic view of biological processes within organoid models. For example, an in vivo xenotransplantation platform using vascularized human brain organoids investigated functional human microglia within a physiologically relevant microenvironment [39]. This study employed a multifaceted approach to validate the physiological resemblance of organoid‐derived microglia to their human counterparts. This included detailed morphological analysis confirming highly ramified structures with complex branching patterns akin to those in human brain tissue. Transcriptomic profiling via single‐cell RNA sequencing demonstrated the acquisition of core microglial gene signatures. Functional validation of organoids is assessed through analytical techniques like liquid chromatography–mass spectrometry and enzyme‐linked immunosorbent assay, which quantify metabolite and protein secretion, respectively. Furthermore, functional assays, such as drug susceptibility testing and the measurement of transepithelial electrical resistance, evaluate their pharmacological responses and barrier integrity. These combined analyses confirm that the organoids accurately replicate key physiological functions of the native tissue.

### 2.3 Organoid Application

The applications of organoids across biomedical research mainly encompass disease modeling, drug screening, mechanistic investigation, and precision medicine [40]. Patient‐derived organoids (PDOs) are 3D microtissues grown in vitro from patient biopsies that can reconstruct phenotypic and genetic characteristics of the original tissue or organ [41, 42]. In disease modeling, PDOs recapitulate pathophysiological features of various disorders, providing a highly relevant human model system [43, 44]. For instance, a comprehensive organoid biobank derived from pediatric kidney cancers was established to include Wilms tumor, malignant rhabdoid tumor, and renal cell carcinoma [45]. The organoids replicated key histological, genetic, and transcriptional features of the original tumors and maintained cellular heterogeneity. The development of PDOs begins with the mechanical and enzymatic disaggregation of primary tissue [46]. The resulting cells are then suspended within an ECM‐rich hydrogel and cultured in a growth factor‐supplemented medium. This process enables the self‐organization of tumor cells into 3D, in vivo‐like structures through cell–cell interactions [47, 48]. These structures closely mimic the histology of original tumor, facilitating sustained cellular expansion and long‐term propagation [49]. The utilization of multiple PDOs could accelerate drug discovery by enabling the identification of promising drug candidates for specific diseases at early stages [50]. Moreover, the establishment of biobanks containing organoids from patients with a wide range of disorders offers an exceptional repository for devising personalized regenerative therapies. The observed correlations between drug responses in PDOs and clinical outcomes in patients with cancers can further inform clinical decision‐making [51, 52]. For drug screening, PDOs offer a more physiologically accurate platform than traditional 2D cultures, enabling high‐throughput compound testing with improved predictive value for drug efficacy and toxicity [53, 54].

## 3 Current Status of Organoids in Cancer Research

**FIGURE 3:**
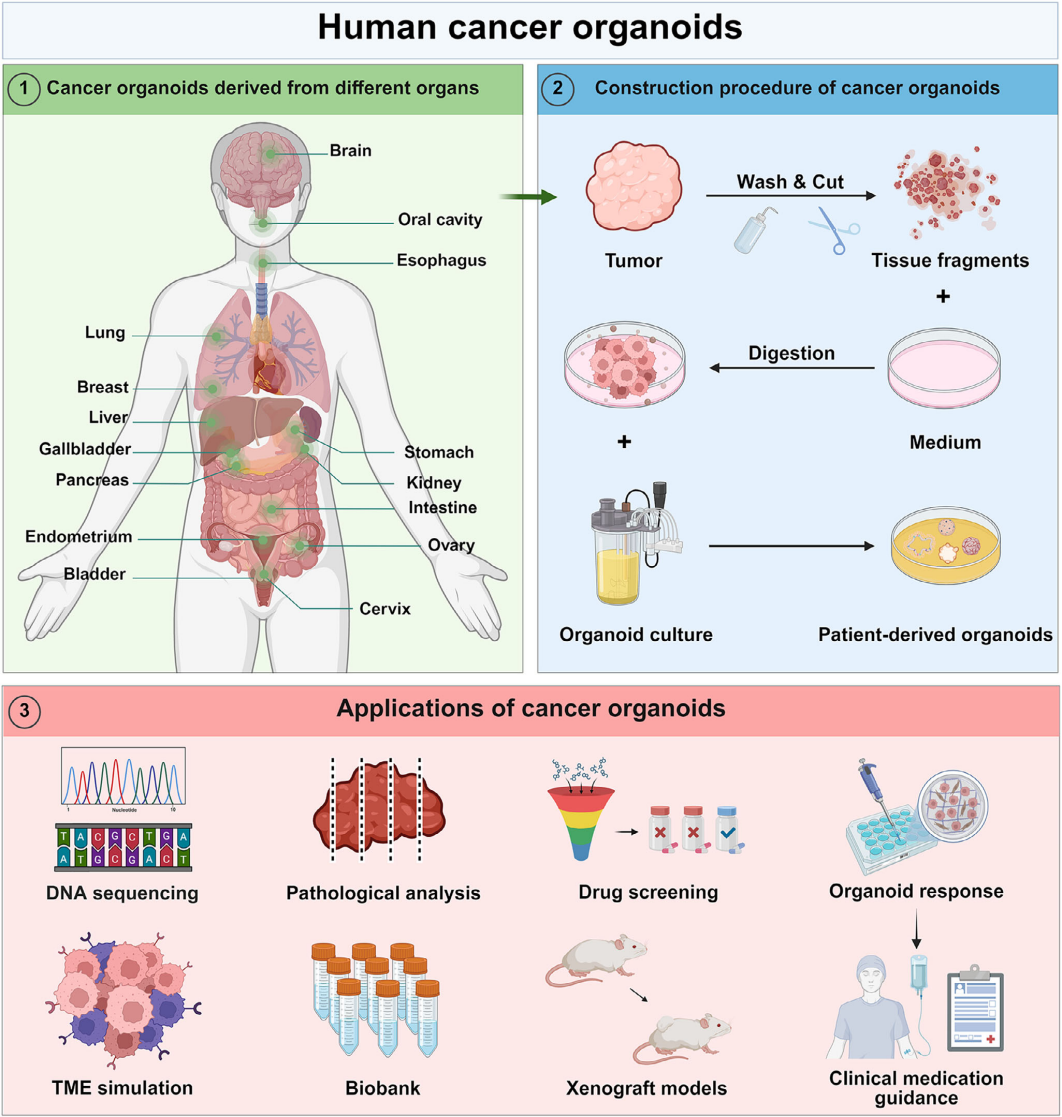
Schematic diagram showing the origin, construction, and applications of human cancer organoids. Through procedures such as tumor resection, washing, and digestion, PDOs are formed under suitable culture conditions and used for further research. Currently, cancer organoids are mainly used for TME simulation, biobank establishment, drug screening, and clinical medication guidance. Created using BioRender.com.

The limited success in isolating pure cancer cell monocultures from patient tissues across diverse tumor types underscores the urgent need to establish cancer organoid models [55]. Cancer organoids are now widely employed across different cancer research areas, including disease modeling, precision oncology, and tumor mechanism analysis (Table 1) [56]. Cancer organoids can faithfully recapitulate the entire spectrum of tumor development from early lesions to advanced metastatic progression [57]. This approach enables the investigation of tumor spatiotemporal heterogeneity and the exploration of interactions in complex TME, which is facilitated by coculture models incorporating stromal and immune cells [58, 59]. In precision oncology, patient‐derived tumor organoids (PDTOs) serve as powerful tools for personalized therapy [60]. High‐throughput platforms utilizing PDTOs systematically assess the sensitivity of chemotherapeutic, targeted, and immunotherapeutic regimens to predict patient‐specific responses to guide clinical treatment decisions [61]. For drug resistance‐related studies, long‐term exposure experiments combined with multiomics analysis elucidate underlying molecular mechanisms and identify effective combination therapies to overcome resistance [62, 63, 64]. Furthermore, integrating PDTO drug sensitivity data with molecular profiling accelerates the discovery of predictive biomarkers for treatment response and prognostic stratification [65]. In cancer biology research, organoid models integrated with clustered regularly interspaced short palindromic repeats (CRISPR)‐associated protein 9 (Cas9) gene editing allow precise characterization of disease‐causing genes, such as the phenotypic consequences of oncogenic mutations or tumor suppressor loss [66]. Additionally, organoid models can mimic the invasive TME, enabling mechanistic studies of metastatic cancer (Figure 3) [67, 68].

**TABLE 1:** Research progress of various cancer organoids.

Organoid	Organoid construction	Application	References
Lung cancer organoid	By using lung adenocarcinoma PDOs, an in vivo metastasis model was established to preserve the biologic characteristics of tumor metastases.	This in vivo PDO metastasis model serves as a versatile platform for studying tumor evolution, evaluating drug efficacy, and developing personalized immunotherapy strategies for lung adenocarcinoma.	[68]
	The model was built by establishing a gel–liquid interface coculture system using lung cancer organoids and their paired peripheral blood mononuclear cells.	Organoids were applied to precisely predict patient responses to anti‐PD1 immunotherapy, dissect tumor–immune interactions via multiomics, and identify potential biomarkers for treatment efficacy.	[69]
	NSCLC organoids were established from patient surgical tissues and patient‐derived xenograft models under optimized culture conditions enabling both short‐term and long‐term expansion.	Organoids were applied for drug testing with targeted therapies and for biomarker validation.	[70]
	Lung cancer specimens from sputum and circulating tumor cells were collected to create a living biobank consisting of 43 lines of PDTOs.	Organoid screening demonstrated that NKX2‐1 expression status determines Wnt dependency and predicts therapeutic response to Wnt‐targeting therapy in lung adenocarcinoma.	[71]
	The organoid testing platform was constructed by integrating a dual‐functional microfluidic chip that combines rapid EGFR mutation detection using a DNA‐based nanoruler and real‐time viability monitoring via a DNA nanosensor.	The platform was used to guide personalized therapy by first rapidly identifying a patient's EGFR mutation status and then selectively testing the corresponding targeted drugs or chemotherapy on their organoids within the same automated system.	[72]
	40 SCLC PDO lines were established, which predominantly carried TP53 and RB1 alterations and were classified into neuroendocrine and non‐neuroendocrine subtypes based on transcriptome profiling.	These organoids were used to identify subtype‐specific growth dependencies, revealing that non‐neuroendocrine‐type SCLCs rely on IGF‐1‐driven YAP1/AP1 signaling, and to validate the efficacy of therapeutic targeting of this pathway.	[73]
	The advanced vascularized lung cancer model was constructed by including patient‐derived lung cancer organoids, lung fibroblasts, and perfusable vessels using 3D bioprinting.	The assessment of drug responsiveness in this model facilitated determination of the appropriate therapy for lung cancer patients with fibrosis.	[74]
Gastric cancer organoid	A biobank of 63 primary GC organoids was established from normal, dysplastic, cancerous, and metastatic tissues from 34 patients, encompassing major molecular subtypes.	The biobank was used for large‐scale drug screening to identify unexpected drug sensitivities and serves as a resource for studying cancer biology and precision therapy.	[64]
	57 GC PDOs were developed from patient tissues, which retained the histological features of the original tumors.	These PDOs were applied to assess individual chemosensitivity, identify gene expression biomarkers predictive of drug response, and validate the results through PDO xenograft models, demonstrating high concordance with clinical outcomes.	[75]
	Docetaxel‐resistant and ‐sensitive GC organoids were created and subjected to single‐cell RNA sequencing to uncover resistance‐associated cellular and molecular alterations.	These organoids were used to identify key resistance genes (FOS, IFI27, PTTG1IP) and validate their functional role in mediating docetaxel resistance.	[76]
	A biological library was established comprising genetically engineered gastric organoids with various GC mutations and 37 PDO lines, including rare genomically stable GCs.	The organoid library was used for phenotype‐based genetic screening to identify mechanisms of niche independency, and to validate the therapeutic potential of Wnt‐targeting therapy through xenografting.	[77]
	Human gastric organoids with biallelic TP53 inactivation were generated and subjected to long‐term experimental evolution to model the earliest stages of gastric tumorigenesis.	This platform was used to trace the evolutionary dynamics of premalignancy, revealing predictable patterns of genomic instability and clonal selection that expose the constraints and barriers to malignant transformation.	[78]
Pancreatic cancer organoid	Patient‐derived conditionally reprogrammed cell lines were transitioned from 2D to 3D organoid cultures using a Matrigel‐based platform without specialized medium to preserve intrinsic molecular subtypes.	3D organoids were used for drug sensitivity profiling, demonstrating superior clinical response prediction compared with 2D cultures by more accurately modeling structural drug resistance barriers.	[32]
	A FPCO was developed by coculturing patient‐derived cancer cells with human iPSC‐derived mesenchymal/endothelial cells and further incorporated THP‐1‐derived macrophages to create M0‐FPCO.	The M0‐FPCO model was used to recapitulate tumor‐associated macrophages diversity and functions, revealing their roles in promoting angiogenesis and enhancing PDAC cell proliferation through single‐cell and bulk RNA sequencing analyses.	[79]
	A high‐throughput T cell‐incorporated pancreatic tumor organoid model was developed by coculturing multicomponent tumor organoids (epithelial, endothelial, fibroblast, macrophage) with tumor‐specific T cells using a two‐step packaging method.	This model was used to screen for epigenetic inhibitors that synergize with anti‐PD‐1 therapy, successfully identifying ITF2357 and I‐BET151 as candidates that reverse immunosuppression and enhance T cell activity.	[80]
	FPCOs were generated by coculturing patient‐derived PDAC cells with human iPSC‐derived mesenchymal and vascular endothelial cells at an air–liquid interface.	These FPCOs were used to model two distinct PDAC states: quiescent (drug‐resistant) and proliferative (recurrence‐prone), serving as a platform for studying drug resistance and screening anticancer therapies.	[81]
	Pancreatic ductal and early cancer organoids with defined genetic backgrounds (KRAS‐G12D ± p53 knockout) were adapted to grow in an acidic extracellular environment (pH 6.7) to mimic tumor niches.	These acid‐adapted organoids were used to investigate how tumor acidosis confers chemoresistance. It was demonstrated that acid adaptation enhances viability and increases the expression of gemcitabine resistance genes, particularly in p53 wild‐type organoids.	[82]
	A panel of eight fully characterized PDAC PDOs was established and analyzed using an AI‐driven live‐cell imaging platform.	These organoids were applied to map intrapatient response heterogeneity at single‐organoid resolution, identifying resistant and invasive clones, and correlating in vitro drug sensitivity with clinical progression‐free survival.	[83]
	A library of 39 patient‐derived PDAC organoids was created and classified into three functional subtypes based on their Wnt and R‐spondin niche factor dependencies.	This organoid library was used to identify the nongenetic mechanisms of niche independence, revealing its association with GATA6‐mediated transcriptional subtypes and tumor progression through genetic perturbation and CRISPR–Cas9 editing.	[84]
	A high‐throughput screening platform was established using a series of isogenic murine pancreatic organoids with defined PDAC driver mutations (e.g., Kras^G12D^) to model both classical and basal phenotypes.	The platform was used to screen over 6000 compounds, identifying perhexiline maleate as an effective agent that selectively targets KRAS‐mutant organoids by suppressing the SREBP2‐cholesterol synthesis pathway.	[85]
	A large‐scale biobank of 260 pancreatic cancer organoid lines was established and characterized through multiomics profiling and therapeutic sensitivity assessments.	This organoid biobank was used to identify novel drivers and biomarkers, uncover chemoresistance mechanisms, and validate statins as a potential therapy, findings which were subsequently translated into a promising clinical trial.	[86]
Hepatobiliary cancer organoid	A biobank of 399 primary liver cancer organoids from 144 patients was established to recapitulate the histological and genomic diversity of the original tumors.	This biobank was used for comprehensive pharmacogenomic profiling to identify predictive biomarkers, uncover c‐Jun‐mediated lenvatinib resistance mechanisms, and screen a synergistic combination therapy.	[65]
	Primary liver cancer organoids from hepatocellular carcinoma, cholangiocarcinoma, and combined tumors were established using a near‐physiological culture system that maintains original tumor characteristics.	These organoids were utilized for biomarker identification, drug screening, and xenograft studies, leading to the discovery of ERK inhibitor SCH772984 as a potential therapeutic agent.	[87]
	Cholangiocarcinoma organoids were integrated with decellularized native tumor or liver scaffolds to create a model that recapitulates the tumor‐specific ECM environment.	This scaffold‐based organoid model was used to study ECM‐tumor interactions, revealing that tumor scaffolds induce a more in vivo‐like transcriptome, enhance chemoresistance, and drive specific stromal reactions compared with standard cultures.	[88]
	Stable, long‐term organoid lines were established from diverse biliary tract cancers to faithfully replicate the histological and molecular features of the original tumors.	These organoids were utilized to identify SOX2 as a prognostic biomarker and to screen a drug library, which revealed the antifungal drugs amorolfine and fenticonazole as potential repurposed therapeutics.	[89]
	Intestine and hepatocyte organoid models were constructed as microtissues to assess nanomedicine toxicity and a PDO model was used for anticancer evaluation.	These organoids were applied to evaluate the biocompatibility of metal‐organic frameworks and to validate the anticancer efficacy of a methotrexate‐loaded nanomedicine (MIL‐125–PEG–MTX).	[90]
Colorectal cancer organoid	CRC organoids were cultured in a customized growth factor‐reduced medium containing FGF10, A83‐01, SB202190, gastrin, and nicotinamide to better maintain original tumor features.	These organoids were used to model tumor characteristics and assess clinical treatment responses.	[91]
	PDOs were generated from metastatic lesions of colorectal cancer patients in a prospective clinical study.	PDOs were specifically used to identify patients nonresponsive to irinotecan‐based chemotherapy, which demonstrated high predictive accuracy for preventing ineffective treatment.	[92]
	A chemically defined PDO system was established to enable long‐term expansion of CRC cells while preserving fetal‐like transcriptional features.	This organoid model identified and characterized an oncofetal state linked to metastasis and therapy resistance, revealing FGF2–AP‐1 signaling as its key regulatory mechanism.	[93]
	Gastrointestinal cancer organoids were established from patients and analyzed alongside liquid biopsy (cfDNA) data capturing mutational profiles.	These organoids were used for functional drug testing of PI3K/mTOR inhibitors, identifying subtype‐specific sensitivities and validating PIK3CA mutations as a potential biomarker for targeted therapy.	[94]
	A large biobank of colorectal cancer PDOs and matched healthy mucosa samples was established for high‐throughput screening.	The biobank was used to screen over 500 bispecific antibodies, leading to the identification of MCLA‐158, which is a therapeutic that selectively eliminates LGR5+ cancer stem cells while sparing healthy cells and inhibits metastasis.	[95]
	A living organoid biobank was generated from patients with locally advanced rectal cancer enrolled in a Phase III clinical trial of neoadjuvant chemoradiation.	These organoids were used to predict patient responses to chemoradiation with high accuracy, demonstrating their potential as a companion diagnostic tool.	[96]
	A biorepository of 65 rectal cancer PDOs was established from primary, metastatic, and recurrent tumors, preserving the molecular traits of the original lesions.	These PDOs were used to model patient‐specific chemotherapy and radiation responses ex vivo and to study invasive and metastatic behavior upon endoluminal engraftment in mice.	[97]
	A CRC organoid–stroma biobank was established by coculturing PDTOs with matched CAFs from 30 patients.	This biobank was used to study stromal‐induced transcriptomic fidelity, identify subtype‐specific drug resistance mechanisms, and screen for targets to overcome stromal‐mediated resistance.	[98]
	CRC organoids were cultured using a novel photocrosslinkable composite bioink compatible with 3D bioprinting, maintaining high viability and structural polarity.	The bioprinted organoid model was applied for high‐throughput drug evaluation, successfully validating clinically used CRC therapeutics.	[99]
Gynecological cancer organoid	A precision cancer care platform was established by creating a living biobank of PDTOs and PDX models, complemented by whole‐exome sequencing data.	The platform performed high‐throughput drug screening on these organoids to identify effective therapies for patients with advanced disease, successfully discovering novel drug candidates validated in PDX models.	[100]
	A novel high‐grade serous ovarian cancer organoid system was developed from patient samples, preserving the native immune microenvironment and vascular components (CD34+ endothelial cells).	These organoids were used to assess cisplatin sensitivity in treatment‐resistant patients and to study associated molecular pathways, providing a platform for evaluating immunotherapy and antiangiogenesis strategies.	[101]
	An optimized method was developed to establish high‐grade serous ovarian cancer organoids from cryopreserved tumor samples, significantly improving culture success rates and enabling long‐term expansion.	These organoids, particularly those maintained in human plasma‐like medium, were used to model patient‐specific drug responses and were provided as a publicly available biobank for translational research.	[102]
	Endometrial cancer PDOs were developed from eight high‐risk patients for experimental modeling.	PDOs were used to validate the therapeutic potential of ASOs targeting SNORD14E, demonstrating their efficacy in inhibiting tumor growth and elucidating the associated RNA regulation mechanism.	[103]
	A protocol was utilized to establish 56 ovarian cancer organoid lines from 32 patients, encompassing major subtypes and recapitulating tumor heterogeneity.	These organoids were applied in drug screening to model subtype‐specific chemotherapy responses and chemoresistance, and were xenografted for in vivo validation.	[104]
	A long‐term 3D organoid culture protocol was developed for cervical epithelia, enabling the establishment of a tumoroid biobank from Pap brush material that retains original HPV genomes.	These organoids were used to study HPV‐associated carcinogenesis, assess differential chemotherapeutic responses, and model xenograft growth.	[105]
	A long‐term 3D organoid culture protocol was established to generate a biobank of cervical pretumoroids and tumoroids from patient samples, retaining original tissue characteristics and HPV genomes.	These organoids were used to evaluate differential chemotherapeutic responses, model xenograft growth, and serve as a platform for screening HPV‐specific T cell responses and therapeutic vaccines.	[106]
	Eight PDO lines were established from three SCCOHT patients to recapitulate the genomic and transcriptomic features of the original tumors.	PDOs were utilized in drug screening to identify methotrexate as a potent and selective therapeutic agent, revealing its mechanism of action through TP53 pathway activation and apoptosis induction.	[107]
Bladder cancer organoid	A biobank of bladder cancer PDOs was established from biopsies, efficiently capturing tumor heterogeneity and enabling interconversion with xenograft models.	PDOs were used to study tumor evolution, correlate drug responses with genomic profiles, and investigate treatment resistance mechanisms.	[63]
	A machine‐learning framework was developed by leveraging network‐based analysis of pharmacogenomic data derived from 3D organoid culture models.	This framework was applied to identify and validate transcriptomic biomarkers that accurately predict patient responses to cisplatin in bladder cancer.	[108]
	Bladder cancer PDOs and normal organoids with ARID1A depletion were established and analyzed alongside public datasets to identify dysregulated molecular effectors.	These PDOs were used for ex vivo drug testing, identifying CHEK1 and BIRC5 as therapeutic targets and validating ARID1A protein expression as a predictive biomarker for treatment response.	[109]
Prostate cancer organoid	Prostate cancer organoid lines were established long‐term from patient biopsies and circulating tumor cells using a 3D culture system, capturing main molecular subtypes.	These organoids recapitulated the genomic landscape of prostate cancer and serve as a versatile platform for genetic and pharmacological studies.	[110]
	Organoids were generated from needle biopsies of metastatic lesions from four NEPC patients, maintaining the molecular features of the original tumors.	These organoids were utilized to elucidate EZH2's role in NEPC progression and for high‐throughput drug screening, identifying potential repurposed therapies for this rare cancer.	[111]
Breast cancer organoid	A direct coculture breast cancer organoid model was developed by sequentially culturing breast cancer cells with TAMs within alginate cryogel‐based ECM.	The model was used to study TAM‐driven enhancement of tumor aggressiveness and identify key signaling pathways and targets through transcriptomic analysis.	[112]
	Organoids derived from a HER2+ breast cancer mouse model (TetO–CMYC/TetO–Neu/MMTV–rtTA) were used to generate single‐cell strand‐seq libraries for multiomics analysis.	These organoids enabled the study of doxorubicin‐induced DNA damage, revealing a significant increase in structural variants and sister chromatid exchanges across different cell types, thus modeling therapy‐driven genomic evolution.	[113]
	A biobank of over 100 primary and metastatic breast cancer organoid lines was generated to recapitulate the histological and molecular diversity of the disease.	This biobank was utilized for in vitro drug screening, demonstrating consistency with patient responses and xenograft models.	[114]
	A biobank of PDXs and matched PDX‐derived organoids was established from treatment‐refractory and metastatic breast cancers.	These matched PDX and organoid models were leveraged for parallel drug screening and real‐time precision oncology, successfully identifying an FDA‐approved drug that induced a complete clinical response in a refractory triple‐negative breast cancer patient.	[115]
Nervous system cancer organoid	Glioblastoma PDOs were cultured in 4D cell‐culture arrays fabricated from thermo‐responsive shape memory polymers.	This platform enabled rapid assessment of drug sensitivity, on‐target activity, and synergistic drug combinations.	[116]
	Glioblastoma PDOs were generated and biobanked using optimized methods that rapidly preserve tumor heterogeneity and molecular profiles.	PDOs were applied to model tumor infiltration, correlate mutational profiles with drug responses, and evaluate CAR‐T cell immunotherapy.	[117]
	Chordoma PDOs were developed from seven tumor samples, recapitulating the histological and molecular heterogeneity of the original tumors.	These PDOs were used in high‐throughput drug screening to identify effective targeted agents (e.g., PI3K/mTOR inhibitors) and to nominate vulnerable pathways for combination therapy development.	[118]

*Abbreviations*: AI, artificial intelligence; ARID1A, AT‐rich interaction domain 1A; ASOs, antisense oligonucleotides; BIRC5, baculoviral IAP repeat containing 5; CAF, cancer‐associated fibroblasts; CAR‐T, chimeric antigen receptor T cell; CHEK1, checkpoint kinase 1; CRC, colorectal cancer; CRISPR–Cas9, clustered regularly interspaced short palindromic repeats‐associated protein 9; ECM, extracellular matrix; EGFR, epidermal growth factor receptor; FDA, Food and Drug Administration; FGF, fibroblast growth factor; FPCO, fused pancreatic cancer organoid; GC, gastric cancer; HER2, human epidermal growth factor receptor 2; HPV, human papillomavirus; IGF‐1, insulin‐like growth factor 1; KRAS, Kirsten rat sarcoma viral oncogene homolog; NEPC, neuroendocrine prostate cancer; NSCLC, non‐small‐cell lung cancer; PDAC, pancreatic ductal adenocarcinoma; PDO, patient‐derived organoid; PDTO, patient‐derived tumor organoid; PDX, patient‐derived xenograft; PI3K/mTOR, phosphatidylinositol 3‐kinase/mammalian target of rapamycin; SCCOHT, small cell carcinoma of the ovary, hypercalcemic type; SCLC, small‐cell lung cancer; SOX2, SRY‐box transcription factor 2; TAMs, tumor‐associated macrophages; YAP1/AP1, Yes‐associated protein 1/activator protein 1.

### 3.1 Lung Cancer Organoid

Lung cancer is clinically classified into small‐cell lung cancer (SCLC) and non‐small‐cell lung cancer (NSCLC). NSCLC dominates both incidence and global mortality [119]. In recent years, considerable progress in lung cancer management has been achieved, which is inseparable from organoid research [69]. For example, Shi et al. engineered patient‐derived NSCLC organoids to expedite target discovery and deepen mechanistic insight into the disease [70]. In this study, these long‐term PDTOs retained histopathology and lineage markers of parental tumors. PDTOs replicated clinically relevant drug responses such as Kirsten rat sarcoma viral oncogene homolog (KRAS)‐mutant models showing mitogen‐activated protein kinase kinase (MEK) inhibitor sensitivity and synergistic fibroblast growth factor receptor (FGFR)/MEK inhibition in FGFR1‐amplified lung squamous cell carcinomas. Through multiomics and phenotypic analyses, Ebisudani et al. uncovered that NKX2‐1 expression delineates Wingless‐related integration site (Wnt)‐dependent from Wnt‐independent states in lung adenocarcinoma, offering an NKX2‐1‐guided stratification framework to forecast the efficacy of Wnt‐directed therapies [71]. A dual‐functional microfluidic chip was developed to integrate rapid epidermal growth factor receptor (EGFR) mutation detection and PDTO drug testing [72]. Cancer cells from NSCLC tissues were dissociated and processed through a DNA‐based nanoruler to detect EGFR mutations within 2 h. Remaining cells were cultured for 7–10 days to form PDOs. Based on the EGFR result, either EGFR‐tyrosine kinase inhibitors or chemotherapy drugs were tested on PDOs. A DNA‐based ATP nanosensor enabled nondestructive, real‐time monitoring of PDO viability during drug response assays by sensing intracellular ATP levels. In the field of SCLC, by establishing a comprehensive library of 40 SCLC PDTOs, researchers identified that non‐neuroendocrine subtypes depend on insulin‐like growth factor 1 (IGF‐1) via activation of the Yes‐associated protein (YAP)–activator protein 1 (AP1) axis, while neuroendocrine subtypes are IGF‐1 independent [73]. Genetic ablation of TP53 and RB1 in human alveolar organoids replicated this IGF‐1 dependency, revealing a lineage‐specific vulnerability. The findings highlighted IGF‐1R inhibition as a potential therapeutic strategy for non‐neuroendocrine‐type SCLC.

### 3.2 Gastric Cancer Organoid

Gastric cancer (GC) constitutes the fifth most common malignancy globally in both incidence and mortality, with approximately one million new cases diagnosed annually, resulting in over 650,000 deaths worldwide [120]. Beyond conventional treatments, management increasingly incorporates minimally invasive surgical techniques for operable disease and integrates immune checkpoint inhibitors with targeted systemic therapies for advanced stages [121]. Future research advancements emphasize the role of organoids in precision medicine, continued innovation in immunotherapy, and multidisciplinary care coordination. Chemosensitivity profiling of PDOs offers a powerful screening platform for selecting optimal chemotherapeutics in GC patients [75]. Drug responses obtained from PDOs were confirmed in PDO‐derived xenograft mice and showed strong concordance with the clinical outcomes observed in GC patients. Through single‐cell RNA sequencing, organoids also enable the identification of cellular and molecular alterations in GC patients, thereby facilitating the discovery of potential therapeutic targets involved in drug resistance [76]. A study established a biobank of 37 GC PDOs encompassing diverse histological and molecular subtypes [77]. Comprehensive characterization revealed distinct genetic and epigenetic mechanisms conferring niche independency, notably identifying cadherin‐1/TP53 compound mutations as drivers of R‐spondin independency. Furthermore, the research demonstrated the efficacy of Wnt‐targeting therapy against Wnt‐dependent GCs using xenografted organoids. This organoid library provided a functional platform for genotype‐phenotype correlation and preclinical drug testing in GC. Karlsson et al. designed a platform that revealed deterministic, tissue‐specific evolutionary trajectories and phenotypic convergence in premalignancy, thus suggesting predictability in early tumorigenesis [78]. CRISPR–Cas9‐engineered biallelic TP53 inactivation in human GC organoids modeled occult gastric preneoplasia over 2 years. TP53 loss triggered progressive aneuploidy, including GC‐prevalent copy number alterations and structural variants acquired in defined temporal orders. High‐throughput lineage tracing showed that rare subclones with shared transcriptional profiles repeatedly achieved dominance through stringent selection and clonal interference.

### 3.3 Pancreatic Cancer Organoid

Pancreatic cancer remains one of the most lethal malignancies, with limited therapeutic options [122]. The characteristically low cellularity of primary tumors necessitates precise molecular characterization [123]. Particularly in pancreatic ductal adenocarcinoma (PDAC), the most common form of pancreatic cancer, the TME is highly complex and populated by diverse stromal cells. To better understand intercellular communications, many studies have turned to cancer organoids as experimental models that recapitulate key features of human PDAC [79, 80, 81, 82]. Concurrently, through systematic analysis of PDO models, integrating molecular profiles, transcriptomic data, and drug response profiles, researchers can now identify potential biomarkers to develop treatment strategies [83, 84, 124]. Consequently, PDOs represent a promising model system for elucidating pancreatic cancer heterogeneity and advancing precision medicine approaches. In 2014, Boj et al. pioneered a method for rapid generation of patient‐derived pancreatic organoids from resected tumors and biopsy specimens [125]. These PDTOs replicated the multistage progression of pancreatic cancer, evolving from early‐grade neoplasms to locally invasive and metastatic carcinomas. Transcriptomic and proteomic profiling identified key genes and pathways dysregulated during disease advancement. Duan et al. developed an isogenic murine pancreatic cancer organoid platform incorporating common PDAC driver mutations [85]. Using this platform for high‐throughput screening of about 6000 compounds, they identified perhexiline maleate as a selective inhibitor of KRAS^G12D^ mutant organoids. Mechanistic studies further revealed that mutant KRAS upregulated the cholesterol biosynthesis pathway via sterol regulatory element‐binding protein 2 (SREBP2). Perhexiline maleate specifically targeted this dependency by inhibiting SREBP2‐mediated cholesterol synthesis and suppressing growth in KRAS mutant PDAC organoids. A comprehensive biobank of 260 pancreatic cancer organoids was established with multiomics profiling [86]. Interestingly, elevated protein glycosylation and cholesterol metabolism were linked to chemoresistance. It demonstrated that statins reverse resistance by inhibiting these pathways and suppressing epithelial‐to‐mesenchymal transition. A Phase 2 clinical trial showed that atorvastatin combined with chemotherapy yielded a response in 70.3% of patients with advanced PDAC, highlighting its potential for overcoming chemoresistance.

### 3.4 Hepatobiliary Cancer Organoid

Hepatobiliary cancers, including hepatocellular carcinoma (HCC), cholangiocarcinoma (CC), and gallbladder carcinoma, rank as the third leading cause of cancer mortality worldwide, accompanied by a steady rise in incidence [126, 127]. Broutier et al. first developed long‐term, genetically stable organoid cultures derived from human primary liver cancers, including HCC, CC, and combined HCC/CC. This platform identified the extracellular signal‐regulated kinase (ERK) inhibitor SCH772984 as a potent therapeutic candidate, validated both in vitro and in vivo via xenograft studies [87]. Since then, biomedical utility of hepatobiliary cancer‐derived organoids for modeling liver cancer biology and advancing personalized medicine has emerged [88, 89, 90, 128]. To facilitate liver cancer precision therapy, Yang et al. established a large biobank of 399 tumor organoids from 144 liver cancer patients [65]. Comprehensive pharmacogenomic profiling revealed extensive genomic and phenotypic intratumor heterogeneity, which correlated with poor prognosis and lenvatinib resistance. They identified c‐Jun overexpression as a key mediator of lenvatinib resistance via JNK and β‐catenin signaling. By linking lenvatinib with a c‐Jun inhibitor, veratramine, a novel compound PKUF‐01 was synthesized and demonstrated synergistic efficacy in resistant organoids. Thus, this study found a lenvatinib‐resistant mechanism for combination therapy. While hepatobiliary cancer organoids hold great potential for personalized medicine, improving establishment efficiency and addressing biological limitations are important for clinical translation [129].

### 3.5 Colorectal Cancer Organoid

Colorectal cancer (CRC) ranks as the third most commonly diagnosed cancer globally, with incidence rates rising notably in emerging economies [130, 131]. Current research advances focus on precision oncology targeting molecular alterations, the application of circulating tumor DNA, and gut microbiome analysis [132]. As a powerful tool to investigate the mechanisms of CRC development, CRC organoids have been widely used in cancer research [91, 92, 133, 134]. Especially for CRC PDOs, they can faithfully recapitulate the diverse histopathological subtypes and clinical stages, differentiation capacity, and molecular profiles of parental tumors in vitro and in xenografts [93, 135, 136, 137]. This preservation enables functional drug testing and biomarker identification for personalized therapeutic design [94, 95, 138, 139, 140]. A living biobank comprising 3D organoid cultures derived from tumor and matched normal tissues of 20 CRC patients was established [141]. These organoids demonstrated compatibility with high‐throughput drug screening, revealing clinically actionable gene‐drug associations. For instance, RNF43‐mutant organoids exhibited hypersensitivity to Wnt secretion inhibitors, TP53 wild‐type organoids showed responsiveness to mouse double minute 2 homolog inhibition, and KRAS mutations conferred resistance to EGFR inhibitors. Another study established a living biobank of PDOs from 80 treatment‐naive locally advanced rectal cancer patients undergoing neoadjuvant chemoradiation [96]. The responses of organoids to irradiation and chemotherapeutic agents in vitro were highly predictive of patient clinical responses to chemoradiation. An orthotopic in vivo model created by engrafting PDOs into the murine rectum replicated tumor invasion and metastasis patterns observed clinically [97]. These findings strongly support the utility of PDOs as a companion diagnostic tool for investigating CRC biology and personalizing CRC treatment. PDTO cultures were also revealed to suppress gene expression programs involved in immune communication and inflammation compared with in vivo tumors [142]. Specifically, introducing monocyte‐derived macrophages into tumoroid cocultures demonstrated that carcinoma cells themselves are sufficient to instruct macrophages to acquire a protumorigenic, immunosuppressive secreted phosphoprotein 1+ (SPP1+) state. Furthermore, SPP1+ macrophages promoted epithelial‐mesenchymal transition in cancer cells expressing CD44. This organoid culture system mapped interactions between CRC cells and the TME in human patients and mouse models. Additional study demonstrated that coculture of PDTOs with cancer‐associated fibroblasts (CAFs) better simulates clinical drug responses and reveals subtype‐specific resistance patterns [98]. Therefore, drug responses derived from coculture models possess prognostic value. Meanwhile, the TME plays an important role in maintaining CRC subtype characteristics and drug responses. This confirms that organoid–stroma coculture models can more accurately reflect in vivo conditions and hold meaningful implications for personalized medicine.

### 3.6 Gynecological Cancer Organoid

Gynecological cancers mainly include ovarian cancer, cervical cancer, and endometrial cancer. These are the three most prevalent malignancies of the female reproductive system. Together, they inflict a heavy burden on women's health, emerging as a worldwide public health issue [143]. Nowadays, cancer organoid models provide a valuable resource for both basic research and personalized medicine in gynecological cancer [100]. This research platform enables drug screening, modeling of chemoresistance, and investigation of tumor evolution [101, 102, 103, 144]. A comprehensive biobank of ovarian cancer PDOs could fully recapitulate the histological, genomic, and transcriptomic diversity of primary tumors across multiple subtypes, including high‐grade serous, endometrioid, clear cell, and mucinous carcinomas [104]. The organoids maintained intratumoral heterogeneity, patient‐specific drug responses, and could be genetically manipulated and xenotransplanted. Few human‐derived models accurately replicate both cervical cancer and the underlying human papillomavirus (HPV) infection. To address this gap, Lõhmussaar et al. devised a long‐term culture protocol that yields stable 3D organoids of ecto‐ and endocervical epithelia [105]. From routine Pap brush specimens, a compact biobank of PDOs was assembled that retained the causative HPV genomes. One isolate harbored the rarely studied HPV30 subtype, which hints at its possible role in carcinogenesis. Similarly, a protocol for precancerous high‐grade squamous intraepithelial lesions was described [106]. When HPV peptide antigens were used to stimulate peripheral blood immune cells cocultured with the organoids, the ensuing virus‐specific T cell responses confirmed that these organoids are robust, reliable platforms for both dissecting anti‐HPV immunity and screening therapeutic HPV vaccines. Furthermore, organoid models represent a valuable tool for discovering targeted therapies for rare cancers. A notable example is small cell carcinoma of the ovary, hypercalcemic type (SCCOHT), a highly aggressive malignancy that predominantly affects adolescent and young adult females and has limited treatment options. Through medium‐throughput drug screening of 153 clinical compounds, methotrexate was identified as a highly potent and selective agent against SCCOHT PDTOs [107]. These results supported a compelling rationale for further clinical investigation of methotrexate as a potential treatment for SCCOHT.

### 3.7 Bladder Cancer Organoid

Bladder cancer ranks as the fourth most common malignancy among men, accounting for approximately 6% of new cancer diagnoses and 4% of cancer‐related deaths [145]. Its incidence is expected to continue rising. This disease exhibits distinct molecular subtypes and pathogenic pathways that diverge significantly between nonmuscle‐invasive (NMIBC) and muscle‐invasive (MIBC) forms [146]. Consequently, effective clinical management necessitates a multidisciplinary strategy integrating both patient‐specific factors and tumor molecular characteristics. PDO has become a powerful model for bladder cancer research [147]. Lee et al. established a biobank of 22 PDO lines from NMIBC to MIBC. These PDOs faithfully recapitulated the histopathological and molecular spectrum, including common mutations like FGFR3, TP53, and FGFR3‐transforming acidic coiled‐coil containing protein 3 (TACC3) fusions of parental tumors [63]. Orthotopic xenografts derived from PDOs maintained tumor features and allowed high‐fidelity interconversion. Drug screening in PDOs revealed responses partially correlating with mutational profiles and treatment history, including resistance in recurrent disease models. Key in vitro responses were validated in matched xenografts, supporting PDOs as models for tumor evolution and precision drug testing. A network‐based machine learning framework integrated pharmacogenomic data from 3D bladder cancer organoid models with protein–protein interaction networks to identify predictive biomarkers for anticancer drug response [108]. The method successfully predicted patient survival following cisplatin treatments, which outperformed conventional models. Through integrative analysis of PDOs and AT‐rich interaction domain 1A (ARID1A)‐depleted normal urothelial organoids, Scholtes et al. found that ARID1A deficiency in bladder cancer upregulated DNA repair and cell cycle‐related genes, particularly checkpoint kinase 1 (CHEK1) and baculoviral IAP repeat containing 5 (BIRC5) [109]. Pharmacological inhibition of both genes selectively induced DNA damage and apoptosis in ARID1A‐deficient tumoroids. ARID1A protein expression may serve as a predictive biomarker for patient stratification toward these targeted therapies.

### 3.8 Prostate Cancer Organoid

Prostate cancer now claims 14.2% of all male cancer diagnoses worldwide, ranking second in incidence and fifth in mortality among men [148]. Prostate cancer organoids serve as a versatile platform for dissecting prostate biology, enabling investigations of tissue homeostasis, tumor initiation and progression, as well as high‐throughput drug screening [110, 149, 150]. The 3D organoid models of advanced prostate cancer were successfully generated from metastatic biopsies and circulating tumor cells of seven patients [110]. These organoids faithfully replicated key genomic features of clinical disease, including transmembrane protease, serine 2–ETS‐related gene (ERG) fusions, speckle‐type POZ protein mutations, serine peptidase inhibitor, Kazal type 1 overexpression, chromodomain helicase DNA‐binding protein 1 loss, and mutations in forkhead box A1, phosphoinositide‐3‐kinase regulatory subunit 1, and DNA repair/chromatin modifier pathways. Critically, all castration‐resistant prostate cancer‐derived lines exhibited biallelic loss of phosphatase and tensin homolog and frequent inactivation of TP53 and RB1 pathways. This approach yielded an extensive panel of patient‐derived prostate cancer models that are adaptable to both genetic and high‐throughput pharmacologic research. To model rare neuroendocrine prostate cancer (NEPC), PDOs from metastatic biopsies were established [111]. The models were utilized to investigate the role of enhancer of zeste homolog 2 (EZH2) in sustaining NEPC‐associated transcriptional programs, and this approach identified both single‐agent and combination therapies with potential efficacy.

### 3.9 Breast Cancer Organoid

Globally, breast cancer represents approximately 30% of all new cancer diagnoses in women and accounts for nearly 15% of female cancer‐related mortality [151]. With incidence rates continuing to rise worldwide, breast cancer persists as a major global health challenge. Therefore, a comprehensive understanding of its complex pathogenesis is essential for developing effective therapeutic interventions. Organoids generated from human breast tissue constitute a powerful model system for investigating breast cancer biology and pathology with a high degree of patient specificity and experimental control [112, 113, 152]. A protocol developed robust long‐term culture conditions for human breast cancer organoids by incorporating Neuregulin 1 into the medium [114]. It generated a living biobank of more than 100 primary and metastatic breast cancer organoid lines representing diverse histological subtypes, grades, and receptor statuses. Breast cancer organoids enabled high‐throughput drug screening, with in vitro responses correlating with in vivo xenograft results and patient treatment outcomes. This resource provides a physiologically relevant preclinical model for studying breast cancer heterogeneity and personalized therapy. Guillen et al. established a large biobank of patient‐derived xenografts and matched organoids from aggressive, treatment‐resistant, and metastatic breast cancers [115]. It is worth noting that they developed an organoid‐based drug screening platform validated by concordant in vivo patient‐derived xenograft responses. As a clinical practice in precision oncology, organoid screening identified eribulin as highly effective in a triple‐negative breast cancer model. Guided by these results, clinical administration of eribulin achieved a complete response and significantly prolonged progression‐free survival in the matched patient.

### 3.10 Nervous System Cancer Organoid

Glioblastoma represents the most prevalent and lethal primary malignant brain tumor in adults, for which no curative therapy exists [153]. Despite advances in elucidating the molecular pathogenesis of glioblastoma, this knowledge has yielded remarkably few approved therapeutic agents. This gap between the urgent clinical need and the persistent failure to translate discoveries into effective treatments underscores the imperative for innovative research platforms capable of transforming the therapeutic development paradigm [116, 154]. Patient‐derived glioblastoma organoids generated directly from fresh tumor tissue without single‐cell dissociation were cultured in a defined, serum‐free medium without exogenous growth factors [117]. Functionally, these organoids exhibited aggressive infiltration upon orthotopic transplantation into mouse brains and retained driver mutations. The organoid model enabled rapid testing of personalized therapies. Organoids showed heterogeneous responses to radiation or temozolomide, targeted drugs such as gefitinib, trametinib, and everolimus correlating with their mutational profiles, and chimeric antigen receptor (CAR) T cell immunotherapy targeting endogenous EGFR variant III. This resource provides a clinically relevant platform for basic research and personalized therapeutic screening in glioblastoma. The first PDTOs for chordoma, a rare and indolent cancer, were established from seven tumor samples [118]. These organoids retained key histological and molecular characteristics of the original tumors. High‐throughput drug screening revealed phosphatidylinositol 3‐kinase (PI3K)/mammalian target of rapamycin (mTOR), EGFR, and Janus kinase/signal transducer and activator of transcription 3 inhibitors as promising therapeutic agents. This finding supports the utility of PDTOs for personalized drug testing and the development of combination therapies for this chemoresistant cancer.

## 4 Current Status of Organoids in Regenerative Medicine

The primary objective of regenerative medicine is to restore normal body function by leveraging biological principles to engineer or regenerate human cells and tissues. Over the past two decades, regenerative medicine has increasingly turned to organoids as an experimental and therapeutic platform [155, 156]. This trend is largely driven by the concept that dissociated stem cells can repair damaged tissues and organs by differentiating into one or more specialized cell types required for functional restoration [157]. The applications of organoids in regenerative medicine involve many aspects. For genetic disorders, developmental abnormalities, and degenerative diseases, organoid culture aims to faithfully recapitulate disease pathophysiology to investigate human development, tissue damage, and repair mechanisms [158]. To facilitate precise drug testing and treatment regimen design, organoid technology enables the assessment of therapeutic efficacy and prediction of organ‐specific toxicity, thereby mitigating risks associated with preclinical animal testing and clinical trials. In tissue engineering and transplantation, organoids serve as a seed cell source for organ regeneration. By constructing structurally complex organoid assemblies that better mimic native human tissues, superior outcomes in replacement therapies can be achieved. These are often complemented by advanced technologies like 3D bioprinting. Here, we provide a detailed summarization of the current applications of organoids in regenerative medicine, spanning from disease understanding to therapy development and tissue engineering (Table 2).

**TABLE 2:** Research progress of organoids in regenerative medicine.

Disease	Organoid construction	Application	References
Intestinal epithelial damage	Intestinal organoids were derived from biopsy samples of patients with CD.	These organoids were analyzed to identify dysregulated peroxisome number and cellular cholesterol as novel disease markers and potential therapeutic targets.	[159]
	A biobank of CD PDOs was created from colonic biopsies of 53 subjects.	These PDOs were used to identify molecular disease subtypes and conduct drug screens to reverse subtype‐specific phenotypes for personalized therapeutics.	[160]
	Clonal human colon organoids were established from patients and analyzed through whole‐exome sequencing.	They were used with CRISPR screening to identify IL‐17 signaling mutations that confer apoptosis resistance, revealing a pathogenesis mechanism in colitis.	[161]
	Intestinal epithelial organoids were generated from patient biopsies and subjected to multiomics profiling and gene editing.	These organoids were used to identify a key DNA methylation mechanism in CD pathogenesis and to develop a prognostic epigenetic signature.	[162]
Liver injury	A microfluidic multiorganoid system was developed to coculture human iPSC‐derived liver and islet organoids under circulatory perfusion.	This system modeled the human liver–islet axis in type 2 diabetes, which demonstrated mitochondrial dysfunction and tested therapeutic effects of metformin.	[163]
	Liver organoids were generated from directly reprogrammed human hepatocyte‐derived bipotent progenitor cells using a defined chemical cocktail.	These organoids demonstrated superior hepatic function in transplantation, effectively modeled alcohol‐related liver disease, and served for sensitive drug toxicity testing.	[164]
	Human hepatic organoids were genetically engineered with a PKD mutation to model congenital hepatic fibrosis.	Organoids replicated disease‐specific fibrotic pathology and identified PDGFRB inhibitors as potential antifibrotic therapies.	[165]
	Cholangiocyte organoids were cultured in defined viscoelastic hyaluronan hydrogels engineered to mimic stress relaxation properties of liver tissue.	These organoids revealed that YAP signaling and hydrogel mechanics drive bile duct formation, enabling disease modeling and regenerative applications.	[166]
	Hepatic organoids were cultured in dynamic stiffening hydrogels mimicking healthy to fibrotic liver tissue to model nonalcoholic fatty liver disease.	This platform revealed stiffness‐induced lipid accumulation and enabled testing of ROCK inhibitors to disrupt mechanosignaling in disease progression.	[167]
	Functionally mature and self‐renewing human hepatic organoids were generated from PSCs.	Organoids were applied in drug toxicity prediction and disease modeling, such as hepatic steatosis, while maintaining long‐term metabolic and detoxification functions.	[168]
	Human liver organoids were derived from multiple iPSC lines and adapted for both high‐throughput 384‐well screening and liver‐on‐chip culture.	Organoids enabled predictive drug‐induced liver injury risk assessment, mechanistic toxicity studies, and detection of drug synergy.	[169]
	Liver organoids incorporating artificial blood vessels were generated by coculturing human iPSC‐derived liver progenitors with human iPSC‐derived endothelial and smooth muscle cells.	Organoids modeled bile duct development and Alagille syndrome, demonstrating functional rescue in cholestatic injury through TGF‐β/Notch signaling.	[170]
Kidney disease	Kidney organoids were generated from human iPSCs and subjected to HIF‐1α overexpression via plasmid transfection or dimethyloxallyl glycine, treatment to enhance vascularization.	These vascularized organoids modeled cisplatin‐induced kidney injury, demonstrating HIF‐1α’s protective role through upregulation of CD31 and SOD2.	[171]
	Engineered human kidney tissues were created from organoid building blocks derived from iPSCs.	These tissues were transplanted into humanized immune mice to model allogeneic rejection and test immunosuppressive therapy.	[172]
	Kidney organoids were transplanted into chicken embryos to study endothelial cell dynamics and vascular maturation.	This model revealed a vein‐to‐arterial phenotypic shift in endothelial cells, identifying SOX7 and blood flow as key drivers for improving organoid vascularization.	[173]
	Kidney organoids were generated from patient‐derived iPSCs with a WT1 missense mutation and compared with CRISPR‐corrected isogenic lines.	Organoids revealed the mutation's role in disrupting podocyte development, demonstrating its rescue through gene editing.	[174]
	Kidney organoids were differentiated from human iPSCs within tunable GelMA hydrogels engineered to mimic adult human kidney stiffness.	These organoids modeled diabetic kidney disease, demonstrating that matrix stiffness influences podocyte maturation and fibrotic response to TGF‐β injury.	[175]
	Induced nephron progenitor cells were clonally expanded from human PSCs through precise manipulation of p38 and YAP signaling pathways.	These cells generated enhanced kidney organoids for CRISPR screening, disease modeling of PKD, and identification of anticystic compounds.	[176]
	Kidney organoids were cultured on a conductive surface enabling real‐time electrochemical signal detection to monitor cell type composition.	This platform nondestructively assessed kidney‐specific differentiation and detected off‐target cells, which facilitated quality control for drug screening and therapeutics.	[177]
	Kidney organoids were derived from human PSCs using renal decellularized ECM hydrogels to enhance cell‐ECM interactions.	Organoids demonstrated improved renal differentiation and vascularization, serving as advanced models for nephrogenesis and personalized medicine.	[178]
	Hypoxia‐enhanced kidney organoids were generated by coinducing metanephric mesenchyme and ureteric bud‐like progenitors from human PSCs.	These organoids with collecting duct structures showed enhanced cyst formation and drug sensitivity for disease mechanism studies.	[179]
Wound healing	Complex skin organoids were generated from human pluripotent stem cells through stepwise induction of cranial epithelial and neural crest cells via TGF‐β and FGF signaling modulation over 4–5 months.	These self‐assembled skin organoids were used to model human fetal skin development and successfully grafted to reconstitute hair‐bearing skin in nude mice.	[180]
	Skin organoids were created by 3D bioprinting a combination of human keratinocytes, fibroblasts, and endothelial cells using a dual‐photo cross‐linking technique.	The customized 3D‐bioprinted skin organoid was implanted into full‐thickness skin defects in mice, where it accelerated wound healing by promoting in situ regeneration and vascularization.	[181]
Bone defect	Woven bone organoids were developed in a dynamic DNA/GelMA hydrogel engineered to mimic biochemical and mechanical properties of bone ECM.	These organoids demonstrated in vivo self‐adaptive osseointegration and provided a scalable platform for bone regeneration and disease modeling.	[182]
	Callus organoids were produced by differentiating human periosteum‐derived cells into microspheroids that mimic the fracture soft callus.	Callus organoids self‐assembled into large tissues that successfully regenerated critical‐sized bone defects in mice.	[183]
	A biomimetic bone organoid model was created using demineralized bone paper to direct osteoblasts into forming mineralized tissue and acquiring bone lining cell phenotypes.	This model enabled the study of localized bone remodeling mechanisms, revealing how bone lining cells direct osteoclastogenesis through paracrine signaling and direct contact.	[184]
	Bone organoids were fabricated using 3D bioprinting with a GelMA/AlgMA/HAP bioink designed to mimic the bone extracellular matrix.	Bioprinted bone organoids promoted multicellular differentiation and demonstrated enhanced bone repair capabilities.	[185]
Neurodegenerative disorder	Human iPSC‐derived microglia were generated and integrated into midbrain organoids to create a more complete neural model.	These microglia‐containing organoids were used to study neuroinflammation, synaptic remodeling, and neuronal excitability.	[186]
	Midbrain organoids derived from PD patient iPSCs were subjected to an optogenetics‐assisted α‐synuclein aggregation induction system to rapidly model protein pathology.	These organoids enabled a compound screening platform that identified BAG956 as a therapeutic candidate capable of reversing α‐synuclein pathology through enhanced autophagic clearance.	[187]
	Midbrain organoids were generated through 3D differentiation of human floor plate neural progenitor cells, including those carrying LRRK2–G2019S mutations.	These organoids modeled PD pathology and revealed patient‐specific decreases in dopaminergic neurons and neurodevelopmental defects.	[188]
Retinal degeneration	Retinal organoids were generated from chemically iPSCs and demonstrated to contain all major retinal cell types.	Photoreceptors from these organoids were transplanted into mouse models of retinal degeneration, where they integrated into the host retina and improved visual function.	[189]
	Retinal organoids were analyzed using 3D‐printed liquid‐metal microelectrodes to precisely target and record from inner retinal layers.	This platform enabled functional assessment of retinal ganglion cell development and synaptic connectivity.	[190]
	Retinal organoids were generated to regulate organoid size through quick reaggregation and controlled BMP signaling to achieve 100% differentiation efficiency.	These highly reproducible organoids were used to analyze early retinal cell fate specification and establish a reliable model for studying human retinogenesis.	[191]
	Retinal organoids were utilized to model the partitioning of the eye cup by manipulating FGF and Wnt signaling pathways.	These organoids revealed how synergistic FGF and Wnt signaling drives ciliary margin formation, elucidating the regulatory code of vertebrate eye development.	[192]
	Light‐sensitive human retinal organoids were generated and characterized through single‐cell RNA sequencing of over 285,000 cells across multiple developmental stages.	Organoids were used to map disease‐associated gene expression to specific cell types, revealing novel cellular targets for retinal diseases like macular degeneration.	[193]
	Retinal organoids were differentiated from human PSCs to generate cone photoreceptors with mature physiological properties.	These organoids demonstrated functional phototransduction comparable to primate fovea, which validated their potential for cell therapy and disease modeling.	[194]
	Retinal organoid sheets were derived from allogeneic iPSCs for transplantation.	These organoid sheets were successfully grafted into patients with retinitis pigmentosa, which demonstrated long‐term graft survival and potential visual function preservation.	[195]
Cartilage and joint degeneration	Cartilage organoids were derived from iPSCs for allogeneic transplantation.	Cartilage organoids integrated into primate knee chondral defects, promoting cartilage repair and expressing lubricating proteins without immune rejection.	[196]
	COPs were constructed by seeding BMSCs onto RGD‐modified silk fibroin–DNA hydrogel microspheres using microfluidic technology.	COPs significantly enhanced cartilage regeneration in vivo by promoting chondrogenic differentiation through integrin and glycosaminoglycan pathways.	[197]
	Cartilage organoids were 3D‐bioprinted using a DNA–silk fibroin hydrogel system loaded with BMSCs and chondrogenic factors to form millimeter‐scale structures.	These 4‐week matured organoids effectively repaired articular cartilage defects in rats by upregulating hyaline cartilage markers and activating the MAPK signaling pathway.	[198]
Cystic fibrosis	Intestinal organoids were cultured from CF patients and the CFTR locus was corrected using CRISPR/Cas9‐mediated homologous recombination in intestinal stem cells.	The gene‐corrected organoids demonstrated restored CFTR function, thus providing proof of concept for gene therapy in hereditary single‐gene defects.	[199]
	Rectal organoids were derived from individual CF patients and used in a forskolin‐induced swelling assay.	These organoids were applied to prospectively select efficacious treatments by demonstrating that their in vitro drug responses correlated with clinical outcomes.	[200]
	Long‐term expanding human airway organoids were established from broncho‐alveolar resections or lavage material, containing basal, multiciliated, secretory, and club cells.	These airway organoids were used to model cystic fibrosis and enable CFTR function assessment, drug screening, and host‐pathogen interaction studies.	[201]
Alpha1‐antitrypsin deficiency	Long‐term expanding human liver organoids were established from adult bile duct‐derived progenitor cells, maintaining genetic stability.	These organoids were differentiated into functional hepatocytes to model AAT deficiency.	[202]
	Liver organoids were established from patient biopsies with different SERPINA1 genotypes (MM, MZ, ZZ) and characterized for AAT protein aggregation and secretion.	These organoids modeled Z‐AAT deficiency pathology and were used to test drug responses, demonstrating their utility for disease modeling and therapeutic development.	[203]
Wilson disease	Prime editing was developed for use in primary adult stem cell‐derived liver organoids to create precise genetic modifications (ATP7B), including disease‐causing mutations and corrections.	These prime‐edited liver organoids were used to model WD and validate the technology's precision and therapeutic potential through whole‐genome sequencing.	[204]
	Patient‐derived intrahepatic cholangiocyte organoids were established from liver cells to model various inborn errors of metabolism, such as WD.	These organoids were used to conduct functional assays for WD, demonstrating their potential for personalized drug testing and metabolic function studies.	[205]
Polycystic kidney disease	Organoids were established from a specific CD24+ epithelial subpopulation of adult kidney and genetically edited using CRISPR–Cas9 to create PKD1/PKD2 knockout models.	These organoids were used to model PKD cyst formation and validate the therapeutic effect of tolvaptan, demonstrating their utility for disease modeling and drug testing.	[206]
	Thousands of uniform kidney organoids with nephron‐like structures were generated from human PSCs, and PKD1/PKD2 genes were inactivated via gene editing to model disease.	These cystic organoids were utilized in a high‐throughput live imaging drug screen, identifying protein kinase inhibitors that specifically block cyst formation without inhibiting overall growth.	[207]
	Kidney organoids were developed from human PSCs and genetically engineered to model PKD, exhibiting characteristic tubular cysts.	These organoids were used to identify defective autophagy in cystogenesis and validate minoxidil as an effective treatment to attenuate cyst formation in vivo.	[208]

*Abbreviations*: AAT, alpha1‐antitrypsin; BMP, bone morphogenetic protein; BMSC, bone marrow mesenchymal stem cell; CD, Crohn's disease; CF, cystic fibrosis; CFTR, cystic fibrosis transmembrane conductance regulator; COPs, cartilage organoid precursors; CRISPR–Cas9, clustered regularly interspaced short palindromic repeats‐associated protein 9; ECM: extracellular matrix; FGF, fibroblast growth factor; HAP, hydroxyapatite; HIF‐1α, hypoxia‐inducible factor‐1 α; iPSC, induced pluripotent stem cell; MAPK, mitogen‐activated protein kinase; PD: Parkinson's disease; PKD, polycystic kidney diseases; PSC, pluripotent stem cell; RGD, arginine–glycine–aspartic acid; ROCK, Rho kinase; SERPINA, serpin family A; SOD2, superoxide dismutase; TGF‐β, transforming growth factor‐β; WD, Wilson disease; WT1, Wilms tumor‐1; YAP, Yes‐associated protein.

### 4.1 Tissue Repair and Organ Transplantation

**FIGURE 4:**
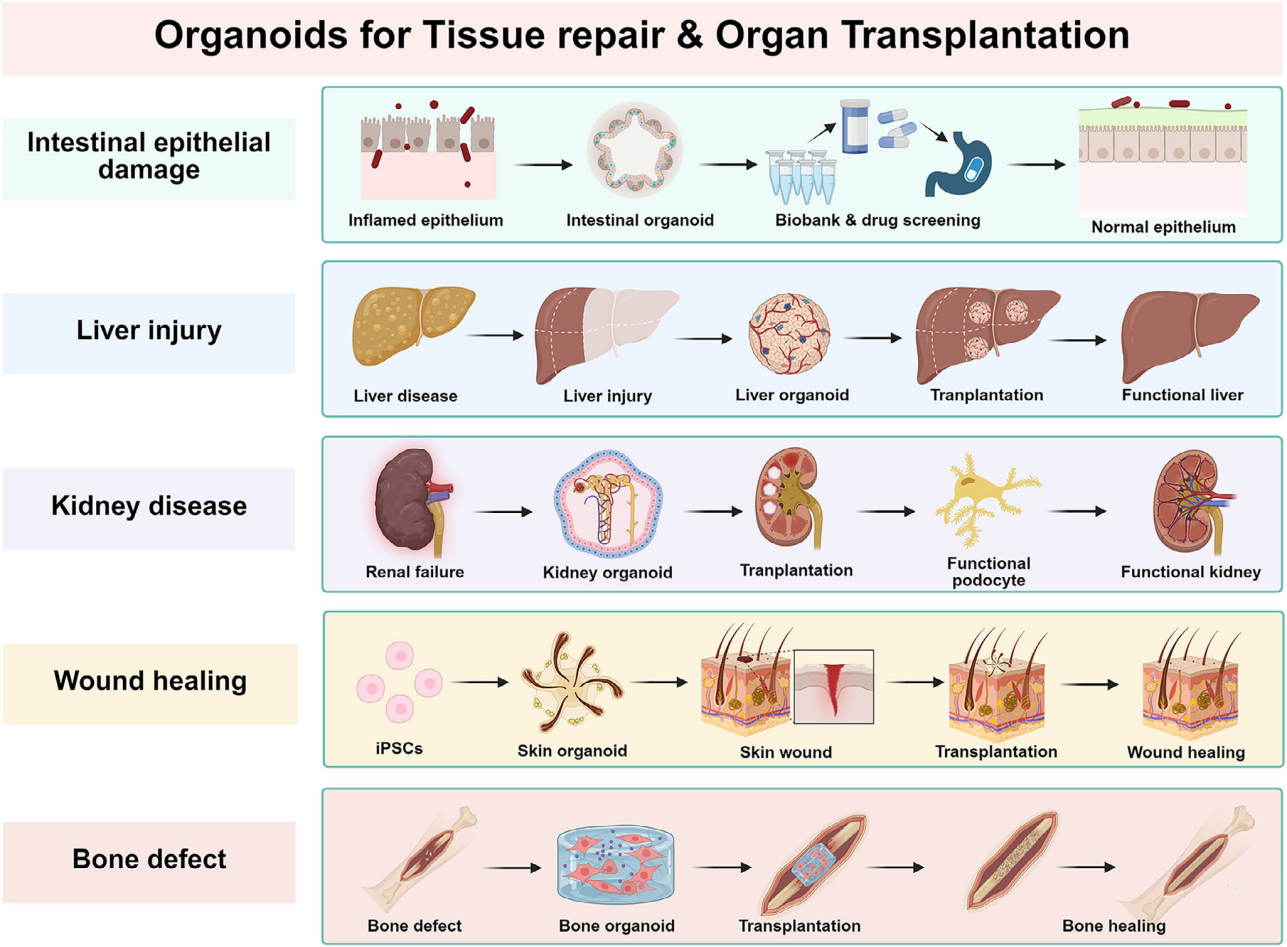
Organoids for tissue repair and organ transplantation. Organoids have demonstrated therapeutic potential in repairing a range of tissue injuries, including those of the intestinal epithelium and liver, as well as in restoring renal function and treating wound and bone defects. Created using BioRender.com.

Current organ transplantation relies largely on donor‐derived organs, which is a solution severely limited by scarcity, immune rejection, and lifelong immunosuppression [209, 210]. Transplantable organoids are now recognized as a promising approach for replacing or regenerating damaged tissues and organs [211]. Given this potential, their ongoing development and scalable manufacturing could yield therapeutic tools that markedly enhance tissue repair (Figure 4) [212].

#### 4.1.1 Intestinal Epithelial Damage

Inflammatory bowel disease (IBD), including Crohn's disease (CD) and ulcerative colitis (UC), is characterized by chronic inflammation that severely compromises the intestinal epithelial barrier, leading to recurrent tissue damage and impaired regeneration [213, 214]. The persistently high rate of drug failure during clinical development is largely attributable to a lack of efficacy and reflects our profoundly limited knowledge of underlying disease pathologies [215]. Intestinal organoids serve as powerful in vitro models to precisely recapitulate this epithelial injury and the ensuing dysfunction mechanisms [159, 216, 217, 218]. For instance, establishing a living biobank of colon PDOs from individuals with IBD could help researchers find distinct molecular subtypes [160]. Since these organoids retained disease‐specific features and responded selectively to subtype‐targeted therapeutics, IBD organoids offer a platform for personalized drug testing and bridge translational gaps in IBD treatment. Nanki et al. demonstrated that chronic inflammation in UC drives somatic mutations in genes involved in interleukin‐17 (IL‐17) signaling, which are distinct from typical CRC drivers [161]. Using clonal organoids and CRISPR screening, they showed that these mutations confer resistance to IL‐17A‐induced apoptosis, promoting selective expansion of mutant epithelial cells. The findings revealed a genetic adaptation mechanism to inflammatory stress and implicated somatic evolution in UC pathogenesis. Based on a large biobank of intestinal epithelial organoids from patients with CD, a study revealed that CD is characterized by stable, region‐specific loss of DNA methylation at major histocompatibility complex (MHC) class I loci, particularly nucleotide‐binding oligomerization domain, leucine‐rich repeat, and CARD domain containing 5 (NLRC5) [162]. Functional validation using gene‐edited organoids confirmed NLRC5 as a key transcriptional regulator of MHC class I and demonstrated its role in exacerbating mucosal inflammation.

#### 4.1.2 Liver Injury

The liver is a multifunctional, structurally complex, and highly vascularized organ composed of numerous microscopic hepatic lobules [219, 220]. Chronic liver disease is responsible for nearly 2 million deaths each year, with key contributing factors including poor diet, high alcohol intake, drug‐induced damage, viral infections, and genetic disorders [221]. The sharp increase in such conditions has resulted in a shortage of donor organs for transplantation [222]. 3D liver organoids have gained prominence as valuable disease models to fulfil liver tissue regeneration needs, as they comprise various hepatic cell types that interact with the surrounding ECM [223, 224]. These features allow them to emulate the biochemical and biophysical properties of native tissue microenvironments [225]. Takebe et al. first succeeded in generating 3D vascularized and functional liver organoids from human iPSCs by transplantation of liver buds created in vitro [226, 227]. The resulting tissue demonstrated high metabolic activity and performed liver‐specific functions, including protein synthesis and species‐specific drug metabolism. This breakthrough established a promising platform for regenerative medicine research. Since then, liver organoid models of metabolic dysfunction‐associated steatotic liver disease, metabolic dysfunction‐associated steatohepatitis, liver fibrosis, cholangiopathy, and type 2 diabetes mellitus have been successively developed [163, 164, 165, 166, 167, 168, 228, 229]. Meanwhile, as an important platform for predicting drug‐induced liver injury, human liver organoids supported high‐throughput screening in 384‐well format [169]. The system demonstrated enhanced albumin secretion, cytochrome P450 activity, and biomarker release in response to hepatotoxins, successfully modeling synergistic toxicity of tenofovir–inarigivir and capturing drug‐specific phenotypic and transcriptomic perturbations. Advanced research focused on integrating human iPSC‐derived blood vessels with liver organoids to model the spatial interaction between portal veins and bile ducts during development [170]. By incorporating immature smooth muscle cells and endothelial cells, the system promoted the differentiation of iPSC‐derived liver progenitors into functional cholangiocytes that form tubular structures exhibiting epithelial features and secretory functions. The model successfully recapitulated transforming growth factor‐β (TGF‐β) and Notch‐mediated signaling pathways essential for bile duct morphogenesis and offers a scalable platform for potential applications in regenerative medicine.

#### 4.1.3 Kidney Disease

Kidneys are vital organs responsible for waste metabolism, electrolyte and fluid balance, and hormone secretion [230]. Kidney disease, such as acute kidney injury, is a frequently observed clinical syndrome characterized by a rapid decline in renal function [231, 232]. This syndrome may progress to chronic kidney disease (CKD) or even end‐stage renal disease (ESRD), which is an irreversible condition associated with high morbidity and mortality [233, 234, 235]. The incidence of CKD and ESRD has risen substantially in recent decades, largely driven by the growing global burden of diabetes and cardiovascular disorders [236, 237]. The advancement of kidney organoid technology now provides a physiologically relevant human model system to pave the way for innovative research [171, 172, 173, 174, 175, 238, 239, 240, 241]. For example, through precise temporal modulation of Wnt and FGF signaling, along with retinoic acid pathway regulation, Takasato et al. realized the directed differentiation of human iPSCs into complex kidney organoids [242]. These organoids contained segmented nephrons with glomeruli, proximal, and distal tubules, and loops of Henle, as well as collecting ducts, endothelial networks, and stromal components. Transcriptomic profiling revealed similarity to the first‐trimester human fetal kidney. A chemically defined culture system enabled long‐term expansion of human nephron progenitor cells, which closely resembled their in vivo counterparts and generated kidney organoids with enhanced maturation, particularly in podocytes [176]. The platform revealed cellular plasticity and facilitated genome‐wide CRISPR screening that identified genes critical for kidney development and disease. Through single‐cell transcriptomic analysis, a specific signaling pathway was identified as a key pathway driving neuronal differentiation [243]. By inhibiting the signaling pathway, 90% off‐target neuronal populations were reduced without impairing renal differentiation, which led to the purification of kidney organoids. Similarly, to minimize off‐target cell populations and enhance the purity of kidney organoids, Suhito et al. established a nondestructive electrochemical monitoring platform to enable real‐time assessment and quality control of kidney organoid differentiation [177]. Moreover, the promotion of vascularization in kidney organoids can be realized by utilizing kidney tissue‐derived decellularized ECM hydrogels to enhance endogenous endothelial differentiation and by developing a coculture system that assembled human PSC‐derived endothelial progenitor spheroids with developing kidney organoids in 3D [178]. Furthermore, kidney organoids derived from human iPSCs serve as effective models for studying drug‐induced nephrotoxicity [244]. Compounds such as doxorubicin and cisplatin have been demonstrated to induce renal damage in organoid models [245, 246].

#### 4.1.4 Wound Healing

Wound healing is a complex biological process that involves a precisely coordinated sequence of cellular and molecular events, including cell migration, proliferation, matrix deposition, and remodeling [247]. In the case of extensive skin wounds caused by trauma, acute illness, or surgical intervention, healing may require several weeks and often results in fibrotic scarring, which can compromise tissue function [248, 249]. As 3D models that emulate human skin, skin organoids have developed into anatomically complex structures. They are now recognized as effective tools for investigating wound healing mechanisms and facilitating the development of wound dressings and skin grafts [250]. Lee et al. established a method for generating complex, hair‐bearing human skin organoids entirely from PSCs by stepwise modulation of TGF‐β and FGF signaling [180, 251, 252]. These organoids developed stratified epidermis, dermis, pigmented hair follicles with sebaceous glands, and functional sensory neural networks. Single‐cell RNA sequencing confirmed their similarity to second‐trimester fetal facial skin. When grafted onto mice, the organoids integrated and formed planar skin with hair growth, demonstrating their potential for modeling skin regeneration.

#### 4.1.5 Bone Defect

A great challenge in orthopedics is the treatment of critical‐sized bone defects. These severe defects most commonly result from traumatic injury, tumor resection, or infection [253]. Based on the diverse components and developmental stages of bone tissue, various bone organoids have been developed to address both physiological and pathological bone defects [254, 255, 256, 257, 258, 259]. Zhu et al. fabricated a dynamic dual‐network hydrogel composed of GelMA and DNA to mimic the mechanical and biochemical properties of native bone ECM [182]. The hydrogel exhibited enhanced viscoelasticity, scavenged reactive oxygen species, and facilitated the self‐organization and mineralization of woven bone organoids. In vivo, organoids demonstrated excellent osteointegration and bone regeneration capabilities. Callus organoids made from human periosteum‐derived cells offer another promising strategy for bone regenerative medicine [183]. These organoids recapitulated endochondral ossification and formed vascularized bone tissue ectopically. When assembled into larger constructs, the organoids bridged critical‐sized long bone defects in mice, which demonstrated structural and functional similarity to native bone. A trabecular bone organoid model using demineralized bone paper replicated the bone remodeling niche [184]. The organoid supported osteoblast mineralization and the acquisition of a bone lining cell phenotype, which responded to chemical stimuli by shifting secretory profiles to promote osteoclastogenesis. Through controlled coculture and spatial mapping, the model demonstrated that paracrine signaling and direct cell‐contact interactions regulate localized bone remodeling, offering a high‐fidelity platform for studying trabecular bone biology and metabolic regulation.

### 4.2 Degenerative Diseases

**FIGURE 5:**
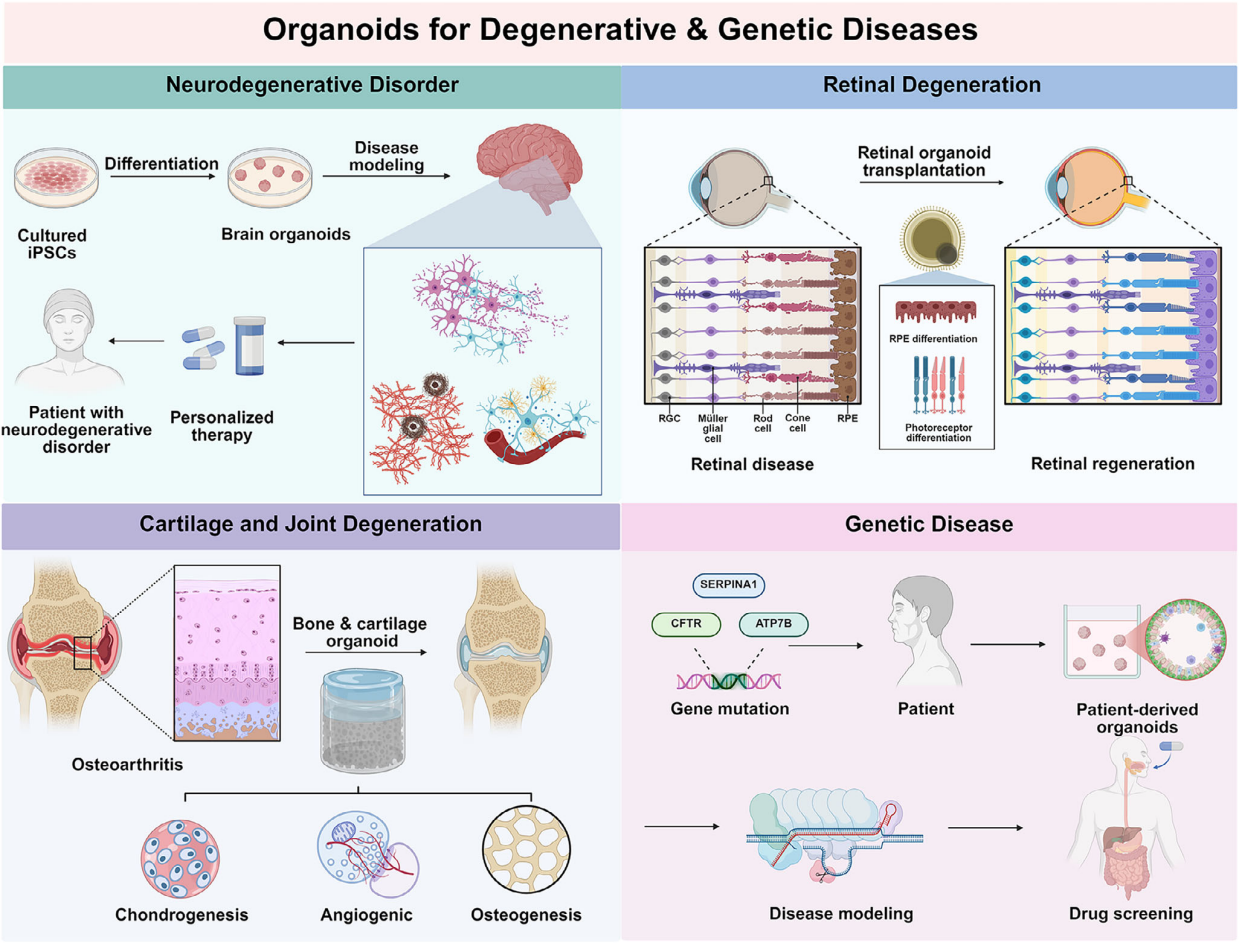
Organoids for degenerative and genetic diseases. For neurodegenerative and genetic disorders, organoids recapitulate disease progression and aid in the development of personalized treatment. In the context of retinal degeneration and joint degenerative diseases, functional organoids promote tissue regeneration and repair. Created using BioRender.com.

By modeling the degeneration of key tissues like the brain, retina, and cartilage, organoids provide deep insights into degenerative diseases. This approach accelerates the discovery of mechanisms and potential regenerative therapies (Figure 5).

#### 4.2.1 Neurodegenerative Disorder

The rise in neurodegenerative disorders caused by diseases such as Alzheimer's disease (AD), Parkinson's disease (PD), and Huntington's disease (HD) has constituted a primary source of global disability [260, 261, 262]. Despite improved outcomes for neurological conditions through preventative strategies and interdisciplinary treatment, combating neurodegenerative diseases requires a deeper understanding of their cause and progression [263]. The advent of human organoid models now allows these complex processes to be examined in a human‐specific context. Human hippocampal organoids (hHOs) derived from iPSCs have emerged as promising models for studying AD, as they recapitulate the neurodegeneration and hippocampal impairments associated with the disorder [264, 265]. To achieve high‐resolution electrophysiological monitoring of hHOs in a noninvasive manner, a novel 3D mesh neurointerface composed of a liquid metal‐polymer conductor was designed [266]. The conductor integrated 128 recording channels and conformed closely to organoid surfaces. Under optimized Wnt and sonic hedgehog signaling, hHOs recapitulated hippocampal development. The system successfully detected neural spikes, synchrony, and glutamine‐responsive activities, offering a powerful platform for modeling hippocampal circuits and neurodegenerative diseases. PD is another common neurodegenerative disorder characterized primarily by movement abnormalities [267, 268]. Since midbrain organoids contain spatially organized dopaminergic neurons, which are particularly relevant to PD pathology, they represent a suitable model for studying the disease [269, 270]. Consequently, efforts have focused on developing patient‐specific midbrain organoids to better model PD [188, 271, 272, 273]. The integration of functionally active, iPSC‐derived microglia into a human midbrain organoid resulted in a more fully functional organoid model that consists of active synapses, astrocytes, and oligodendrocytes [186]. Microglia presence reduced cell death and oxidative stress, enhanced synaptic remodeling, and increased neuronal excitability. The established system provided an advanced platform for modeling PD research. Kim et al. developed an optogenetics‐assisted α‐synuclein aggregation induction system by using human iPSC‐derived dopaminergic neurons and midbrain organoids to model PD pathology [187]. Through high‐content screening of 1280 compounds, BAG956 was identified as a potent inhibitor of α‐synuclein aggregation. The compound demonstrated neuroprotective effects in α‐synuclein preformed fibril models and supported its potential as a therapeutic candidate.

#### 4.2.2 Retinal Degeneration

Retinal degeneration encompasses a group of debilitating disorders, such as age‐related macular degeneration and retinitis pigmentosa, that cause irreversible vision loss through the progressive deterioration of retinal cells, ultimately culminating in complete blindness at advanced stages [274]. In response, many research efforts are directed toward using retinal organoids both to model diseases and to serve as a potential source of donor photoreceptors for cell transplantation therapies [189, 190, 191, 192]. By integrating highly multiplexed protein imaging, single‐cell transcriptomics, and chromatin accessibility data, a multimodal spatiotemporal atlas of human retinal organoid development can now be presented [275]. This provides new insights into retinal cell fate regulation and organoid fidelity to native human retina. Cowan et al. developed light‐sensitive, multilayered human retinal organoids from iPSCs with functional synapses and characterized their cell types using single‐cell RNA sequencing [193]. The transcriptomes of organoid cell types converged toward those of adult peripheral retina. Genes associated with retinal disease, such as retinitis pigmentosa and age‐related macular degeneration, were mapped to show cell‐type‐specific expression patterns conserved in adult retina and developed organoids. This resource enabled modeling of retinal diseases and identified cellular targets for mechanistic studies and therapeutic interventions. For macular degenerative diseases, the dysfunction and eventual death of the cones is the main cause of central blindness. Human PSC‐derived retinal organoids developed cone photoreceptors capable of intrinsic light‐evoked responses without exogenous chromophores [194]. These cones exhibited electrophysiological properties that closely resemble those of adult primate foveal cones. Therefore, the use of retinal organoids can be used as potential sources of functional cones for cell replacement therapies. The first‐in‐human transplantation of allogeneic iPSC‐derived retinal organoid sheets into two patients with advanced retinitis pigmentosa demonstrated long‐term stability without immune rejection or tumorigenesis [195]. Increased retinal thickness was observed at the transplant site. While visual acuity did not improve significantly, one patient showed potential functional gains in light sensitivity. This clinical study proved the feasibility and safety of retinal organoid transplantation for retinal degenerative diseases.

#### 4.2.3 Cartilage and Joint Degeneration

As the most common form of arthritis, osteoarthritis (OA) is a leading cause of chronic pain and long‐term disability in adults [276]. It is a multifactorial disease characterized by pathological changes across the entire joint structure [277]. Among the affected tissues, cartilage plays a pivotal role as a connective tissue that provides structural support and cushioning to maintain normal joint function [278]. In recent years, advances in cartilage organoid development have shown promising potential for promoting cartilage regeneration and effectively slowing the progression of OA [279]. The engraftment of allogeneic iPSC‐derived cartilage organoids in a primate model of articular cartilage defects showed no immune rejection and prevented degenerative changes in surrounding cartilage [196]. Single‐cell RNA sequencing confirmed chondrocyte differentiation and supported the potential clinical application of allogeneic iPSC‐based cartilage repair. Shen et al. developed arginine–glycine–aspartic acid (RGD)‐modified silk fibroin–DNA hydrogel microspheres (RSD‐MS) via microfluidics and photopolymerization to serve as a scaffold for cartilage organoid precursors [197]. RSD‐MS exhibited enhanced chondrogenic differentiation of bone marrow mesenchymal stem cells (BMSCs) in vitro through integrin‐mediated pathways and glycosaminoglycan biosynthesis. To support a sustained chondrogenic differentiation of BMSCs and inhibit fibrosis and hypertrophy, they further developed a DNA–silk fibroin hydrogel sustained‐release system by covalently grafting glucosamine and TD‐198946 [198]. Using digital light processing bioprinting, millimeter‐scale cartilage organoids were fabricated, with 4‐week organoids exhibiting optimal hyaline cartilage phenotypes, demonstrating their potential as an advanced graft strategy for cartilage repair.

### 4.3 Genetic Disease

Since organoids derived from patient‐specific stem cells can recapitulate key features of genetic diseases in vitro, studies have made progress in formulating personalized therapeutic strategies and improving diagnostic accuracy (Figure 5) [199, 280].

#### 4.3.1 Cystic Fibrosis

Cystic fibrosis (CF) is a rare, monogenic hereditary disorder caused by mutations in the CF transmembrane conductance regulator (CFTR) gene, which encodes the CFTR protein [281, 282, 283]. This dysfunction results in defective mucociliary clearance and chronic airway dehydration. With a global prevalence exceeding 100,000 individuals, CF has traditionally been managed through supportive strategies such as nutritional intervention, airway clearance, and antibiotic regimens. Recent progress toward precision medicine in CF has been driven by PDOs that reliably forecast clinical benefit from small‐molecule therapeutics in vitro [284, 285]. For instance, forskolin‐induced swelling in rectal organoids derived from CF patients serves as a robust in vitro biomarker for predicting individual responses to CFTR modulator therapies [200]. The organoid responses showed significant correlation with two key clinical endpoints: changes in lung function and sweat chloride concentration. Particularly for individuals with rare CFTR mutations not covered by standard genotype‐based therapies, this method shows high predictive accuracy. Similarly, by measuring forskolin‐induced swelling and steady‐state lumen area, Dekkers et al. identified responders to ivacaftor even among patients with rare, uncharacterized CFTR mutations [286]. Moreover, long‐term expanding human airway organoids derived from ASCs also serve as versatile models for studying CF through functional CFTR assays [201].

#### 4.3.2 Alpha1‐Antitrypsin Deficiency

Alpha1‐antitrypsin (AAT) deficiency is one of the most common genetic diseases caused by mutations in the serpin family A1 (SERPINA1) gene, which encodes the AAT protein [287]. This condition predisposes individuals to both pulmonary and hepatic impairment. Misfolded AAT proteins accumulate in the endoplasmic reticulum of hepatocytes, promoting protein aggregation that can progress to chronic liver failure. In severe cases, liver transplantation may become necessary. Bipotent stem cells derived from adult human liver bile ducts were used to construct liver organoids that could maintain genomic stability over months of expansion [202]. Following the initiation of hepatocyte differentiation, patient‐specific organoids modeling AAT deficiency exhibited the formation of intracellular AAT protein aggregates and a consequent increase in apoptosis. Building upon the aforementioned study, Gómez‑Mariano et al. further utilized liver organoid models to make fine distinctions between disease subtypes of AAT deficiency [203]. They found that organoids from individuals with homozygous (ZZ) and heterozygous (MZ) genotypes showed impaired expression of hepatocyte markers such as albumin and apolipoprotein B, along with characteristic diastase‐resistant polymer accumulation. Transcriptional analysis confirmed hepatocyte‐specific gene expression upon differentiation, and response to oncostatin M stimulation indicated preserved regulatory mechanisms.

#### 4.3.3 Wilson Disease

Wilson disease (WD) is an autosomal recessive disorder characterized by impaired copper homeostasis, resulting in the toxic buildup of copper in various tissues [288]. This condition arises from mutations in the ATPase copper transporting beta (ATP7B) gene. Copper accumulates predominantly in the liver and brain, potentially leading to hepatic dysfunction, neurological impairments, and psychiatric manifestations. Schene et al. achieved precise genomic corrections in organoid models of WD by using prime editing [204]. Prime editing is a CRISPR–Cas9‐based technology utilizing a nickase‐reverse transcriptase fusion that is expected to correct genetic defects [289]. Prime editing showed higher accuracy and fewer deletions than Cas9‐mediated homology‐directed repair. Whole‐genome sequencing of edited clones revealed no detectable off‐target effects, supporting its potential for therapeutic genome editing in monogenic disorders. Intrahepatic cholangiocyte organoids derived from patients as in vitro models for studying inborn errors of metabolism successfully recapitulated disease phenotypes in WD, demonstrating utility in functional assays related to metal homeostasis [205]. PDOs exhibited greater susceptibility to copper, demonstrating an IC_50_ of 0.28 mM CuCl_2_, which was lower than the 0.33 mM observed in healthy control organoids. However, the model showed variable gene expression and functional maturity, with limitations in representing certain metabolic pathways or severe cellular defects. The potential of WD organoids for personalized drug screening requires further evaluation.

#### 4.3.4 Polycystic Kidney Disease

Polycystic kidney diseases (PKDs) constitute a prevalent etiology of ESRD, characterized by the development of numerous fluid‐filled cysts within the kidneys [290, 291, 292]. The two principal monogenic forms are autosomal dominant PKD and autosomal recessive PKD, both of which are disorders associated with ciliary dysfunction. Currently, there are few effective therapeutic options for PKD. Moreover, the scarcity of accessible patient biopsies complicates direct research into PKD pathogenesis. These problems emphasize the urgent demand for innovative disease models to advance the understanding of disease mechanisms and develop new treatment strategies for PKD. Recently established kidney organoids offer a promising model for studying PKD, as they can spontaneously develop cyst‐like structures in vitro [179, 206, 293, 294]. A scalable platform for generating human kidney organoids with consistent nephron‐like structures further enabled large‐scale disease modeling and drug screening. For instance, by introducing loss‐of‐function mutations in PKD1 and PKD2 via CRISPR–Cas9, Tran et al. established organoid models of autosomal dominant PKD [207]. A high‐throughput phenotypic screen of protein kinase inhibitors identified several compounds, notably quinazoline, which effectively inhibited both cyst initiation and expansion without compromising overall organoid viability. Kidney organoid models derived from human PSCs carried mutations associated with both autosomal dominant and recessive PKD [208]. These organoids recapitulated key pathological features, including tubular cyst formation, injury, and dysregulated renin‐angiotensin‐aldosterone signaling. Through single‐cell multiomics, metabolic dysregulation and impaired autophagy were identified as central to cystogenesis. Genetic restoration of autophagy or ablation of primary cilia suppressed cyst formation, revealing a cilium‐autophagy metabolic axis. An autophagy activator, minoxidil, highlighted its therapeutic potential for PKD by significantly reducing cyst growth in vivo.

## 5 Current Limitations and Challenges

**FIGURE 6:**
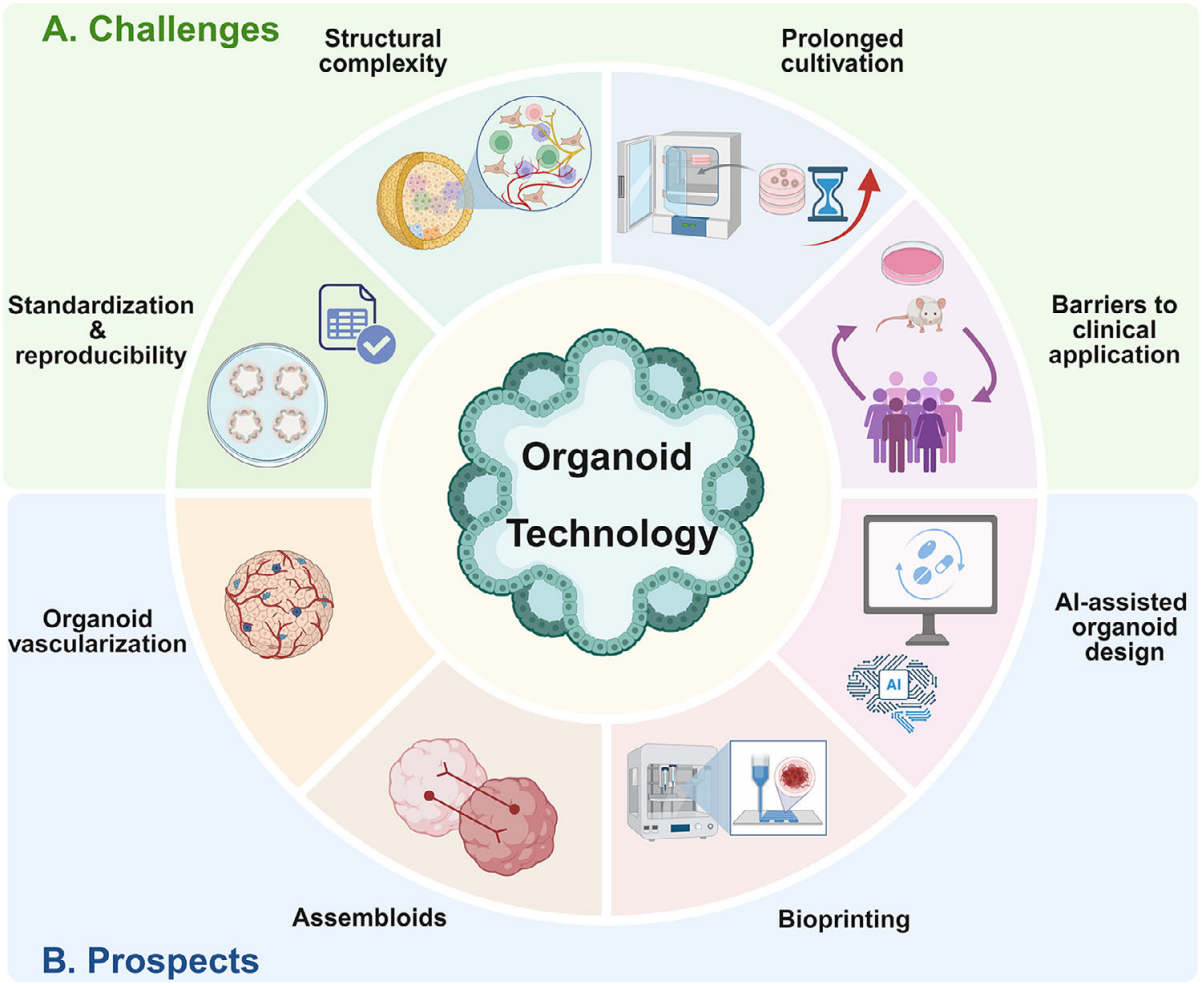
Challenges and prospects of organoid technology. (A) Challenges include organoid standardization, achieving structural complexity, prolonged cultivation, and clinical translation. (B) Prospects include organoid vascularization, assembloid construction, bioprinting technology, and AI‐assisted organoid design. Created using BioRender.com.

Organoids have been extensively utilized in cancer research and regenerative medicine, demonstrating great value and unique advantages, particularly in disease modeling, personalized medicine, and drug discovery. However, current applications of organoids face several challenges that hinder the full potential of this technology. These include standardization, realization of structural complexity, prolonged cultivation periods, and barriers to clinical application (Figure 6A). If future studies can effectively address these challenges, organoids will be better applied in clinical practice.

### 5.1 Standardization and Reproducibility

Variations in organoid culture protocols, culture environments, and operator techniques across laboratories lead to inconsistencies in organoid construction [295, 296]. Furthermore, batch‐to‐batch variability in the quality of matrices like Matrigel contributes to interorganoid heterogeneity, which will impact research outcomes [297, 298]. Therefore, establishing standardized protocols for generating organoids modeling specific diseases is essential. Future efforts should focus on continuously optimizing organoid culture technologies to develop a mature, stable, controllable, and animal‐component‐free culture system. Concurrently, the implementation of a fully automated, standardized organoid production platform may be imperative in the future [299]. Through standardized culture combined with automation, organoids can enable medium‐ to high‐throughput screening [245]. Liu et al. presented a droplet‐based microfluidic system for the high‐throughput generation of highly uniform pancreatic cancer organoids from single cells using defined alginate microgels [300]. This method enabled precise control over organoid size and morphology by encapsulating individual cells in tunable microenvironments with stiffness mimicking native pancreatic tumors. The platform supported scalable production and reproducible drug response testing.

### 5.2 Inadequate Organoid Complexity and Maturity

A primary goal of organoid research is to faithfully replicate the physiological and pathological microenvironment governing cellular growth and behavior in vivo. Consequently, developing diverse and functionally sophisticated organoid models is crucial. However, current organoid development remains relatively simplistic. They often lack essential features such as functional vascularization, innervation, or immune cell infiltration [301, 302, 303]. This limitation restricts the modeling of immunotherapy responses in tumor organoids [304]. Incorporating extra cellular and noncellular elements into organoid cultures elevates their intricacy [305]. Therefore, future research should prioritize enhancing the functional diversity and tissue maturity of organoid systems across various organs [306]. This advancement is expected to minimize the discrepancy between in vitro models and the authentic physiological/pathological in vivo microenvironment. Concurrently, efforts should focus on developing multiorgan chips to simulate interorgan interactions and establishing coculture systems integrating diverse cell types, such as immune cells, neurons, and endothelial cells [307].

### 5.3 Prolonged Cultivation and High Costs

Organoid culture often requires several weeks or even months to develop fully. This prolonged cultivation period compromises research efficiency, making it particularly challenging for long‐term studies or high‐throughput applications such as drug screening. The need for specialized media, growth factors, and advanced bioreactors further escalates the economic and logistical burdens, restricting the scalability and accessibility of organoid technology. Moreover, extended in vitro culture increases the risk of genetic instability, as organoids may acquire mutations or exhibit phenotypic drift over repeated passages [129]. This not only threatens the reproducibility of experimental results but also raises concerns regarding the reliability of organoids in modeling human diseases and predicting clinical outcomes. To address these challenges, several solutions are being proposed, such as implementing standardized transport protocols, creating mutation‐specific media, and leveraging the power of single‐cell sequencing to uncover novel disease mechanisms [308].

### 5.4 Barriers to Clinical Application

While organoids have been widely applied in disease modeling and drug screening, most applications remain confined to basic or preclinical research. Therefore, a critical next step for organoid‐based medicine is the transition from in vitro models to controlled human clinical trials. It is encouraging to observe the increasing incorporation of organoid models into clinical trial designs at some leading hospitals and research centers, especially for oncological applications (Table 3). This reflects a broader shift toward functionally validated, patient‐specific preclinical models in translational research. However, accelerating the clinical translation still needs a coordinated set of actions. These include establishing a regulatory framework for utilizing organoids as drug screening and toxicity testing models or potential therapeutic products [309]. It is also essential to conduct large‐scale prospective clinical studies to validate organoid‐based therapeutic efficacy, correlate in vitro drug sensitivity with clinical treatment responses, and perform cost‐effectiveness analyses [97]. Moreover, developing medical regulatory and ethical guidelines for organoid applications is imperative [310]. Scalable and automated production systems must be developed to meet future clinical and industrial demands [311]. Meanwhile, building an integrated data infrastructure, including unified storage, standardized analytical pipelines, and sharing platforms, will support disease‐specified translational research.

**TABLE 3:** Ongoing clinical trials related to cancer organoids.

Disease	Sponsor	Objective	Study start	Status	NCT No.
Lung cancer	The University of Texas Health Science Center	To assess the predictive power of lung cancer PDOs by establishing a biobank from Stage I–IV patients, and systematically evaluating their ability to recapitulate ex vivo responses to chemotherapeutic and targeted agents	2018‐10‐16	Recruiting	NCT03655015
Lung cancer	Henan Cancer Hospital	To leverage PDOs for both drug sensitivity testing and predictive modeling, with the ultimate goal of translating these ex vivo findings into clinical benefit for lung cancer patients pleural effusion	2025‐02‐21	Recruiting	NCT06959173
Lung cancer	Affiliated Hospital of Jiangnan University	To evaluate the concordance and predictive accuracy of PDOs for patients with lung cancer	2023‐02‐01	Recruiting	NCT05669586
Lung cancer	The First Affiliated Hospital of Soochow University	To assess the antitumor effect of tumor‐reactive T cells in lung cancer PDOs	2025‐07‐10	Active, not recruiting	NCT07239544
Neuroendocrine neoplasms of the gastro‐entero‐pancreatic tract	Regina Elena Cancer Institute	To establish organoids derived from resected tumor tissue and adjacent healthy tissue, following standard clinical protocols	2023‐03‐03	Recruiting	NCT06519500
Gastric cancer	Cancer Institute and Hospital, Chinese Academy of Medical Sciences	To compare the drug screening results from gastric cancer organoid models with the sensitivity to neoadjuvant chemotherapy and immunotherapy agents recommended by clinical guidelines	2023‐05‐01	Recruiting	NCT06196554
Advanced pancreatic neuroendocrine tumor	Ruijin Hospital	To evaluate the treatment efficacy of chemotherapy and targeted therapy regimens when guided by the results of PDO drug sensitivity testing in the context of advanced, unresectable pancreatic neuroendocrine tumors	2024‐04‐03	Recruiting	NCT06246630
Pancreatic cancer	Prof. Dr. med. Dres. h.c. Jan Schmidt, MME, Klinik Hirslanden	To validate PDOs as a predictive biomarker for guiding individualized therapy in metastatic pancreatic cancer by correlating drug response in vitro with specific tumor characteristics	2024‐10	Not yet recruiting	NCT06615830
Hepatobiliary cancer	Fondazione Policlinico Universitario Agostino Gemelli IRCCS	To develop PDOs that recapitulate key features of the in vivo tumor microenvironment and mimic the gut‐liver axis crosstalk to establish correlations with clinical prognosis and to screen the efficacy of existing systemic therapies	2024‐10‐01	Recruiting	NCT06929845
Hepatobiliary cancer	Peking Union Medical College Hospital	To investigate heterogeneous protein characteristics of primary liver cancer organoids using photoacoustic imaging	2024‐08‐01	Recruiting	NCT07101237
Hepatobiliary cancer, colorectal cancer	James Yun‐wong Lau, Chinese University of Hong Kong	To evaluate efficacy of NGS/PDO guided treatment in patients with inoperable or metastatic HCC and colorectal cancer	2024‐10‐18	Recruiting	NCT06077591
Colorectal cancer	Nanfang Hospital, Southern Medical University	To determine whether chemotherapy regimens selected through PDO drug sensitivity testing can improve the outcomes of stage IV colorectal cancer	2023‐05‐01	Recruiting	NCT05832398
Colorectal cancer	Regina Elena Cancer Institute	To generate paired PDOs from primary colorectal cancers and their synchronous liver metastases, and to employ these models for functional analysis of genes regulated by the NF‐Y/p53 complex	2024‐05‐25	Recruiting	NCT06787625
Ovarian cancer	University of Udine	To establish ovarian cancer organoids for delineating the mechanisms by which multicellular crosstalk among tumor cells, immune cells, and the resident microbiota influences tumor biology and therapeutic response	2024‐01‐02	Recruiting	NCT06272240
Endometrial cancer	Regina Elena Cancer Institute	To develop and optimize a standardized workflow for generating endometrial cancer organoids from surgical specimens, including the evaluation of transport conditions to ensure reproducible culture establishment	2025‐03‐23	Recruiting	NCT07258186
Bladder cancer	Qilu Hospital of Shandong University	To utilize PDO models to conduct drug sensitivity testing for intravesical chemotherapy in NMIBC	2024‐10‐01	Recruiting	NCT06662071
Bladder cancer	University of Bern	To apply PDO‐based drug screening to guide the choice of neoadjuvant intravesical therapy (epirubicin, mitomycin C, gemcitabine, or docetaxel) for NMIBC	2024‐10	Recruiting	NCT06227065
Prostate cancer	Tianjin Medical University Second Hospital	To create a prostate cancer organoid‐chip model from clinically obtained tissues of patients with visceral metastases, and to employ high‐throughput drug sensitivity screening on this platform to evaluate common chemotherapy drugs for the selection of optimal individualized treatment plans	2024‐06‐01	Recruiting	NCT06536725
Prostate cancer	Sun Yat‐sen University	To assess the clinical impact and safety of employing PDO drug sensitivity testing to inform treatment decisions in post‐first‐line mCRPC with bone metastases	2024‐07‐24	Recruiting	NCT06529549
Breast cancer	Xiangya Hospital of Central South University	To verify the predictive accuracy, clinical feasibility, and reproducibility of the PDO model, and to establish a standardized framework for evaluating treatment regimens to advance precision medicine in breast cancer	2024‐06‐01	Recruiting	NCT06702800
Breast cancer	Guangdong Provincial People's Hospital	To compare the clinical outcomes of therapy guided by PDO drug sensitivity testing versus physician's choice of treatment in patients with previously treated, HER2‐negative, locally advanced or metastatic breast cancer	2024‐10‐01	Recruiting	NCT06102824
Breast cancer	Second Affiliated Hospital, School of Medicine, Zhejiang University	To evaluate the predictive value of PDOs for chemotherapy and targeted therapy response in metastatic breast cancer, PDO models will be established from hydrothorax/ascites of eligible patients and subjected to drug testing against agents including doxorubicin, carboplatin, cyclophosphamide, paclitaxel, herceptin, and pertuzumab	2024‐11‐09	Recruiting	NCT06658080
Glioblastoma	Centre Henri Becquerel	To establish the feasibility in routine clinical practice of generating ex vivo organoid cultures from perioperative glioblastoma samples, including glioblastoma organoids and blood vessel organoids	2025‐07‐01	Not yet recruiting	NCT07029100
Glioblastoma	Chungnam National University Hospital	To assess the prognostic value of PDOs in predicting therapeutic response to both conventional and repurposed drugs, such as temozolomide, in glioblastoma cases	2021‐08‐18	Recruiting	NCT06782984

*Abbreviations*: HCC, hepatocellular carcinoma; mCRPC, metastatic castration‐resistant prostate cancer; NGS, next‐generation sequencing; NMIBC, nonmuscle invasive bladder cancer; PDO, patient‐derived organoid.

## 6 Conclusion and Prospects

Future directions for organoid development will center on several research fields, most notably organoid vascularization, the construction of assembloids, advances in bioprinting techniques, and artificial intelligence (AI)‐assisted organoid design (Figure 6B).

### 6.1 Organoid Vascularization

Vascularization delivers oxygen and nutrients while removing metabolic waste, thereby sustaining the viability and function of regenerated tissues [312]. Therefore, vascularized organoids can extend the culture period and better mimic vasculature‐related physiological functions. Without perfusion, organoids would experience impaired metabolism, leading to restricted growth, inadequate nutrient supply, and compromised functionality [313]. Recently, there have been breakthrough advancements in the vascularization of organoids. Vascularized lung and intestinal organoids were generated from human iPSCs by codifferentiating mesoderm and endoderm within a single spheroid [314]. Bone morphogenetic protein (BMP) signaling fine‐tuned the endoderm‐to‐mesoderm ratio so that vascularization enhanced organoid maturation, cellular diversity, 3D architecture, and alveolar formation, particularly when combined with bioengineered scaffolds. Saiki et al. generated functional human liver sinusoidal endothelial cell progenitors (iLSEPs) from human PSCs by recapitulating developmental pathways [315]. Using an inverted multilayered air–liquid interface culture system, they cocultured iLSEPs with hepatic endoderm, mesenchymal, and arterial progenitors. This enabled the self‐organization of liver bud organoids containing branched sinusoidal‐like vascular networks expressing key markers. These sinusoids exhibited functional characteristics that were essential for hepatocyte maturation and vascular patterning. In another study, by using micropatterned human PSCs, researchers mimicked the earliest stages of organ‐specific vascularization [316]. A specific cocktail of growth factors and small molecules was identified that, when applied to geometrically confined human PSCs, enabled the formation of cardiac vascularized organoids, which structurally and functionally resembled a 6.5‐week human embryonic heart. The same vascular‐inducing cocktail successfully generated hepatic vascularized organoids, suggesting a conserved developmental program governs vasculogenesis across different organ systems.

### 6.2 Assembloids

Assembloids refer to integrated systems formed by combining distinct types of organoids or specialized cell types to mimic multitissue interactions within the body [317, 318]. By facilitating crosstalk between different lineages, assembloids better recapitulate the complexity of organ communication and systemic physiology in development and disease [319, 320]. For example, region‐specific brain organoids derived from human PSCs can be fused with other organoid types to generate multiregion assembloids. These models are valuable for investigating complex intercellular interactions and the mechanisms of neural circuit formation within the human nervous system [321]. Human floor plate organoids derived from stem cells were combined with spinal cord organoids to generate human midline assembloids [322]. Onesto et al. characterized the secretome of human floor plate cells and identified several proteins that were not expressed in the mouse floor plate. This model provided novel insights into human‐specific aspects of neurodevelopment and midline connectivity. Another function of the assembloid is to provide a research platform for studying disease progression and evaluating drug efficacy. A representative study employed a human iPSC‐derived midbrain–hindbrain assembloid to examine the propagation of α‐synuclein pathology in PD [323]. The hindbrain organoids carrying a synuclein alpha gene triplication recapitulated key pathological features, including elevated phosphorylated and insoluble α‐synuclein. When assembled with healthy midbrain organoids, pathological α‐synuclein spread from the hindbrain to the midbrain region, inducing synaptic alterations and transcriptional changes associated with early disease mechanisms.

### 6.3 Bioprinting

Bioprinting is an advanced additive manufacturing technique that precisely deposits living cells and biomaterials layer‐by‐layer to create bioengineered structures [324, 325, 326]. Utilizing organoids as bio‐inks to fabricate large, structured tissues for transplantation is particularly promising because they contain self‐organizing progenitor and differentiated cells capable of forming functional tissue units [249]. Moreover, organoid‐based bioprinting supports vascularization and functional maturation, which is essential in repairing complex tissue or organs [327]. For instance, to ensure adequate nutrient delivery to all cells and successful integration with the host vasculature, the engineered vascular network must exhibit a high degree of structural organization. Bioprinting offers a way to precisely design and control the initial architecture of such vascular networks [328]. On the other hand, bioprinting allows for the efficient creation of organoid models that accurately mimic specific diseases [99, 329]. By using 3D bioprinting technology, PDTOs, lung fibroblasts, and perfusable vessels were incorporated into a lung cancer model [74]. To more faithfully replicate the native lung tissue, researchers generated a porcine lung‐derived decellularized ECM hydrogel that delivers both physical and biochemical cues to the lung cancer microenvironment. Notably, organoids developing fibrosis exhibited markedly larger shifts in resistance to targeted sensitizing agents when cultured in this hydrogel than in Matrigel. Through the incorporation of compatible biomaterials, bioprinting promotes effective regeneration at the transplantation site. By adapting to the wound microenvironment, extrusion bioprinting technology enables efficient embedding and assembly of spherical skin organoid hydrogels under temperature control, thereby accelerating wound healing [181]. To treat large‐scale bone defects, Wang et al. employed bioprinting technology to mimic the sophisticated structure of native bone tissue [330]. By incorporating bioinks, they engineered a highly complex analog of the bone ECM. The resulting bioprinted scaffolds support the extended culture and gradual maturation of bone organoids, promote multilineage cell differentiation, and yield important insights into the early mechanisms of bone formation [185].

### 6.4 AI‐Assisted Organoid Design

AI is being frequently applied to integrate with organoid technology to enhance the precision, scalability, and functionality of these 3D models [331]. First, AI facilitates the rapid screening of organoid construction strategies to generate more physiologically accurate organoids by optimizing parameters such as matrix material synthesis, spatial structure discernment, cell culture conditions, and identification of growth factors. Second, AI enables cost‐effective extraction of multiscale image features, automating the analysis of morphological, cellular, organoid, and tissue‐level images. Deep learning algorithms further improve the accuracy and efficiency of image segmentation and feature quantification [332]. In addition, AI supports precise preclinical evaluation by predicting drug responses, modeling diseases, and personalizing treatment strategies using PDOs. Therefore, AI‐enabled organoids represent a transformative approach for advancing biomedical research and clinical applications.

## 7 Conclusion

Organoids hold great potential to revolutionize our understanding of human biology and disease modeling, thereby accelerating the development of precision medicine in oncology and regenerative medicine. To fully realize this potential and overcome persistent challenges in scalability, standardization, and functional maturation, a concerted interdisciplinary effort is indispensable. Fostering deeper collaboration among biologists, engineers, and clinicians will be important in translating this promising technology into improvements in human health.

## Author Contributions

Conceptualization: Ruiyang Li, Ke Xu, and Jiacan Su. Original draft writing: Ruiyang Li, Yuezhou Wu, and Zhu'anzhen Zheng. Draft reviewing and editing: Ruiyang Li, Yuezhou Wu, Zhu'anzhen Zheng, and Ke Xu. Investigation: Ruiyang Li, Yuezhou Wu, and Zhu'anzhen Zheng. Project administration: Fengjin Zhou and Ke Xu. Supervision: Jiacan Su. Visualization: Ruiyang Li. All authors have read and approved the final version of the manuscript. Ruiyang Li, Yuezhou Wu, and Zhu'anzhen Zheng contributed equally to this work.

## Ethics Statement

The authors have nothing to report.

## Conflicts of Interest

The authors declare no conflicts of interest.

## Data Availability

The data that support the findings of this study are available in the article.
